# microRNA-27a-3p but Not -5p Is a Crucial Mediator of Human Adipogenesis

**DOI:** 10.3390/cells10113205

**Published:** 2021-11-17

**Authors:** Hang Wu, Taner Pula, Daniel Tews, Ez-Zoubir Amri, Klaus-Michael Debatin, Martin Wabitsch, Pamela Fischer-Posovszky, Julian Roos

**Affiliations:** 1 Department of Pediatrics and Adolescent Medicine, Ulm University Medical Center, 89075 Ulm, Germany; hang.wu@uni-ulm.de (H.W.); taner.pula@uni-ulm.de (T.P.); klaus-michael.debatin@uniklinik-ulm.de (K.-M.D.); Pamela.Fischer@uniklinik-ulm.de (P.F.-P.); 2 Division of Pediatric Endocrinology and Diabetes, Department of Pediatrics and Adolescent Medicine, Ulm University Medical Center, 89075 Ulm, Germany; Daniel.Tews@uniklinik-ulm.de (D.T.); martin.wabitsch@uniklinik-ulm.de (M.W.); 3 Inserm, CNRS, iBV, Université Côte d’Azur, 06103 Nice, France; Ez-Zoubir.Amri@unice.fr

**Keywords:** microRNA-27a, adipocytes, adipogenesis, PPARγ, LPL

## Abstract

MicroRNAs (miRNAs), a class of small, non-coding RNA molecules, play an important role in the posttranscriptional regulation of gene expression, thereby influencing important cellular functions. In adipocytes, miRNAs show import regulatory features and are described to influence differentiation as well as metabolic, endocrine, and inflammatory functions. We previously identified miR-27a being upregulated under inflammatory conditions in human adipocytes and aimed to elucidate its function in adipocyte biology. Both strands of miR-27a, miR-27a-3p and -5p, were downregulated during the adipogenic differentiation of Simpson–Golabi–Behmel syndrome (SGBS) cells, human multipotent adipose-derived stem cells (hMADS), and human primary adipose-derived stromal cells (hASCs). Using miRNA-mimic transfection, we observed that miR-27a-3p is a crucial regulator of adipogenesis, while miR-27a-5p did not alter the differentiation capacity in SGBS cells. In silico screening predicted lipoprotein lipase (LPL) and peroxisome proliferator activated receptor γ (PPARγ) as potential targets of miR-27a-3p. The downregulation of both genes was verified in vitro, and the interaction of miR-27-3p with target sites in the 3′ UTRs of both genes was confirmed via a miRNA-reporter-gene assay. Here, the knockdown of LPL did not interfere with adipogenic differentiation, while PPARγ knockdown decreased adipogenesis significantly, suggesting that miR-27-3p exerts its inhibitory effect on adipogenesis by repressing PPARγ. Taken together, we identified and validated a crucial role for miR-27a-3p in human adipogenesis played by targeting the essential adipogenic transcription factor PPARγ. Though we confirmed LPL as an additional target of miR-27a-3p, it does not appear to be involved in regulating human adipogenesis. Thereby, our findings call the conclusions drawn from previous studies, which identified LPL as a crucial regulator for murine and human adipogenesis, into question.

## 1. Introduction

Obesity is one of the major health problems in the Western world [1]. The excessive accumulation of adipose tissue leads to a state of chronic, low-grade inflammation and is associated with severe comorbidities such as type 2 diabetes mellitus, hepatic steatosis, cardiovascular disease and an increased risk of cancer [2]. On the one hand, white adipose tissue (WAT) can expand due to increased energy intake through the accumulation of lipids in already-existing adipocytes, a process called hypertrophy. On the other hand, progenitor cells within the adipose tissue can differentiate into new, mature adipocytes, a mechanism known as hyperplasia [3]. This storage of excess energy in the form of triglycerides in WAT is required to prevent ectopic fat deposition in the liver and muscles, which is associated with insulin resistance and non-alcoholic fatty liver disease [4]. However, the healthy expansion of WAT is limited, and further challenge with excess nutrients may drive adipocyte hypertrophy, which causes adverse metabolic effects [5]. Therefore, it is of utmost importance to elucidate the processes leading to the formation of new adipocytes from tissue-resident precursor cells. Both events, adipogenesis and lipid accumulation, are known to be regulated by distinct signaling pathways. Key mediators of adipogenesis involve the different transcripts of CAAT/enhancer-binding proteins (C/EBPs) and peroxisome proliferator activated receptor γ (PPARγ), which act in a transcriptional cascade. In addition, insulin, mTOR, Notch and Wnt signaling pathways play pivotal roles in adipogenesis [6,7].

Since the discovery of microRNAs (miRNAs) in 1993 by Victor Ambros’ laboratory [8], several studies have identified miRNAs as important regulators of both adipocyte differentiation and metabolism [9,10,11]. MiRNAs are a class of small, non-coding RNA molecules that are only 18–25 nucleotides long [12]. They play an important role in the regulation of cellular gene expression by either repressing the translation of protein-coding genes or cleaving the target mRNA to induce its degradation [12,13]. MiRNAs are initially transcribed as primary transcripts (pri-miRNAs) by RNA polymerase II (Pol II) and are then cleaved by the endonuclease Drosha, to liberate a hairpin-structured precursor miRNA (pre-miRNA). Pre-miRNAs are exported to the cytoplasm in an Exportin5/RanGTP-dependent manner and are further processed to short double-stranded miRNA duplexes. Next, one of the miRNA strands is loaded onto Argonaute proteins (AGO) and exerts its function in the miRNA-induced silencing complex (miRISC) [14]. In this process, one strand of the duplex is preferentially incorporated, which is named the guide strand, whereas the mainly discarded strand is known as the passenger strand [14]. In some cases, however, both strands of the duplex can be loaded onto the AGO complex in an equal distribution [15]. Other findings suggest that the levels of both strands vary in different tissues [16,17]. Under certain pathological conditions, for example, under changes in target availability, the ratio of the passenger to guide strand can change [18,19]. This strand selection defines the target specificity of the miRISC, thereby providing a switch-like mechanism for altering gene expression [18].

We previously identified miR-27a as being upregulated in an in vitro model of WAT inflammation and were therefore interested in its role in human adipocyte biology [20]. MiR-27a had been identified as a mediator of white adipocyte differentiation, yet those studies did not analyze the differential effects of the guide strand, miR-27a-3p, and the passenger strand, miR-27a-5p. Therefore, we aimed to characterize, in this study, the effects of both miRNA strands, miR-27a-3p and -5p, side by side, on human adipocyte differentiation.

## 2. Materials and Methods

### 2.1. Human Primary Material

Human primary adipose-derived stromal cells (hASCs) were isolated from mammary WAT samples and cultured as described [21,22]. Mammary tissue samples were collected from 9 female patients who underwent plastic surgery. The donors were 45.33 ± 5.43 years old (mean ± SEM), and their BMI was 26.65 ± 1.60 kg/m^2^ (mean ± SEM). All the procedures were authorized by the ethics committee of Ulm University (entry number 300/16). Written informed consent was obtained from all the patients in advance, and all the associated methods were conducted in accordance with approved guidelines for human experimental research.

### 2.2. Cell Culture

Simpson–Golabi–Behmel syndrome (SGBS) preadipocytes [23,24], human multipotent adipose-derived stem cells (hMADS) [25] and hASCs were cultured and differentiated using an adipogenic induction cocktail as described [24]. The adipogenic induction cocktail was FBS-free DMEM-F12 (ThermoFisher Scientific, Germany) supplemented with 10 μg/mL transferrin, 20 nM insulin, 100 nM cortisol, 200 pM T3, 25 nM dexamethasone, 250 μM IBMX and 2 μM rosiglitazone (all from Sigma-Aldrich, Taufkirchen, Germany). Four days later, the medium was changed to FBS-free DMEM-F12 supplemented with 10 μg/mL transferrin, 20 nM insulin, 100 nM cortisol and 200 pM T3.

### 2.3. miRNA Mimic and siRNA Transfection

For miRNA mimic transfection, SGBS preadipocytes or hMADS cells were transfected with 20 nM miR-27a-5p mimic (Syn-hsa-miR-27a-5p, Qiagen, Hilden, Germany), miR-27a-3p mimic (Syn-hsa-miR-27a-3p, Qiagen, Hilden, Germany) or non-target control (AllStars Negative Control siRNA, Qiagen, Hilden, Germany) mixed with 0.66 μL/cm^2^ of Lipofectamine 2000 (Invitrogen, Karlsruhe, Germany) according to the manufacturer’s protocol.

For siRNA transfection, SGBS preadipocytes were transfected with 20 nM LPL-siRNA (siGENOME Human LPL siRNA, Horizon Discovery, Cambridge, UK) or non-targeting control (siGENOME Non-Targeting Control siRNA Pool #2, Horizon Discovery, Cambridge, UK) mixed with 0.66 μL/cm^2^ of Lipofectamine 2000.

### 2.4. Determination of Differentiation Rate

A net micrometer was used to determine the numbers of preadipocytes and mature adipocytes. The differentiation rate was determined from the quotient of mature adipocytes, defined by containing at least five clearly visible lipid droplets, to the total cell number.

### 2.5. Oil Red O Staining

SGBS cells were fixed with 4% PFA for 5 min and subsequently washed with 60% 2-propanol. Afterwards, the cells were incubated with Oil Red O solution for 10 min. The cells were rinsed with dH_2_O, and microphotographs were taken using a BZ-9000 fluorescence microscope (Keyence, Neu-Isenburg, Germany).

### 2.6. Triglyceride Determination

Cellular lipids were extracted by rinsing the cells twice with hexane/2-propanol (3:2), and the solution was evaporated at 30 °C for 45 min using a Concentrator 5301 (Eppendorf, Hamburg, Germany) and stored at −20 °C. For the measurement, the samples were dissolved in 100% 2-propanol, and Triglyceride Reagent, Free Glycerol Reagent, and Glycerol Standard (all from Sigma-Aldrich, Taufkirchen, Germany) were used to determine the triglyceride content according to the manufacturer’s protocol.

### 2.7. RNA Isolation and Reverse Transcription

Cells were harvested with Tri-Reagent (Zymo Research, Freiburg im Breisgau, Germany), and total RNA was isolated with a Direct-zol RNA mini Prep Kit (Zymo Research, Freiburg im Breisgau, Germany) according to the manufacturer’s protocol. The RNA was reverse transcribed with SuperScript II Reverse Transcriptase (Thermo Scientific, Dreieich, Germany).

### 2.8. Quantitative Real-Time PCR (qPCR)

A miScript II RT Kit and miScript SYBR Green PCR Kit (Qiagen, Hilden, Germany) were used for miRNA quantification. Mature miRNAs were quantified by the miScript primer assay for Hs-miR-27a*_1 and Hs-miR-27a_1. The results were normalized to SNORD68_11 (sno68) (all from Qiagen, Hilden, Germany) using the 2^−ΔCt^ method [26].

The mRNA levels were quantified with the SsoAdvanced Universal SYBR Green Supermix on a CFX Connect plate cycler (BioRad, Feldkirchen, Germany) using the primers given below. The results were normalized to HPRT using the 2^−ΔCt^ method [26].

The primer sequences were (5′ > 3′): Adiponectin-FWD: GGC CGT GAT GGC AGA GAT; Adiponectin-REV: CTT CAG CCC CGG GTA CT; FASN-FWD: CTA CCT GAG CAT AGT GTG GAA GAC GCTG; FASN-REV: CAT CCC ACT GGT ACA CCT TCC CAC TCAC; HPRT-FWD: GAG ATG GGA GGC CAT CAC ATT GTA GCC CTC; HPRT-REV: CTC CAC CAA TTA CTT TTA TGT CCC CTG TTG ACT GGT C; LPL-FWD: GTC AGA GCC AAA AGA AGC A,LPL-REV: ATG GGT TTC ACT CTC AGT CC; Perilipin-FWD: GAA GTT GAA GCT TGA GGA GCG AGG ATG G; Perilipin-REV: GGC TTC CTT AGT GCT GGT GTA GGT CTT CTG; PPARγ-FWD: GAC CAC TCG CAT TCC TTT GAC ATC AAG CC; PPARγ-REV: TGA TCG CAC TTT GGT ATT CTT GGA GCT TCA G.

### 2.9. Protein Isolation and Western Blotting

Cells were washed with PBS and harvested using lysis buffer containing 10 mM Tris-HCl, 150 mM NaCl, 2 mM EDTA, 1% TX-100, 10% glycerol supplemented with 1 mM DTT and cOmplete Protease Inhibitor Cocktail (Roche, Mannheim, Germany). After incubating them on ice for 30 min, the lysates were cleared by centrifugation (14,000× *g*, 30 min, 4 °C).

SDS-PAGE was performed with 15 μg of protein using Bolt 4–12% Bis Tris Plus gels (both from ThermoFisher Scientific, Dreieich, Germany) in 1× Bolt MES running buffer. A Trans-Blot Turbo System and Trans-Blot Turbo RTA Mini 0.2 µm Nitrocellulose Transfer Kit (all from BioRad, Feldkirchen, Germany) were used for protein transfer. Images were captured with the ChemiDoc MP imaging system (BioRad, Feldkirchen, Germany) in the corresponding channel.

Primary antibodies: anti-adiponectin (GeneTex, AF1065, Germany), anti-FASN (Cell Signaling, #3180, Frankfurt am Main, Germany), anti-LPL (Santa Cruz Biotechnology, sc-73646, Heidelberg, Germany), anti-perilipin (abcam, ab3526, Cambridge, UK), anti-PPARγ (Cell Signaling, #2443, Germany), and anti-tubulin (Calbiochem, CP06, Frankfurt am Main, Germany).

Secondary antibodies: StarBright™ Blue 520 Goat Anti-Mouse IgG (BioRad, #12005867, Germany), StarBright Blue 700 Goat Anti-Mouse IgG (BioRad, #12004158, Germany), StarBright™ Blue 520 Goat Anti-Rabbit IgG (BioRad, # 12005870, Germany), StarBright Blue 700 Goat Anti-Rabbit IgG (BioRad, #12004162, Germany), and m-IgGκ BP-HRP (Santa Cruz Biotechnology, sc-516102, Germany).

### 2.10. Target Prediction Analysis

To identify potential targets of miR-27a-3p that could explain the observed phenotype, the online available miRNA-target-prediction algorithms miRWalk [27], StarBase [28] and TargetScan [29] were used with default settings with the search term “hsa-miR-27a-3p”. An enrichment analysis with all the potential miR-27a-3p targets represented in all three databases was performed using the online available gene list enrichment analysis tool EnrichR [30] (https://maayanlab.cloud/Enrichr/, accessed on 18 June 2020). Enriched pathways with an adjusted *p*-value <0.01 in WikiPathways 2019 were further analyzed. Finally, the predicted miR-27a-3p targets of the enriched pathway Adipogenesis WP236 were compared to the sequencing data for miR-27a-transfected SGBS adipocytes reported by Galhardo et al. [31].

### 2.11. Dual-Luciferase Reporter Assay

A dual-luciferase assay was performed as described previously [32]. The interaction of miR-27a-3p with the predicted binding sites in the LPL or PPARγ mRNAs was assessed using the pmirGLO Dual Luciferase miRNA target expression vector (Promega, Walldorf, Germany). The predicted binding sites were annotated by TargetScan [29] in the 3′ UTR of the transcript NM_000237 of LPL from base pairs 7319 to 7523, and 3′ UTR of the transcript NM_015869.5 of PPARγ from base pairs 7336 to 7443. Both binding sites were cloned into the 3′ UTR of the firefly luciferase reporter gene encoded on pmirGLO. For the dual-luciferase assays, 25 ng of a dual-luciferase vector containing the predicted binding sites of miR-27a-3p and 100 nM miR-27a-3p mimic or NT siRNA were co-transfected into HEK293 cells using Lipofectamine 2000 (ThermoFisher Scientific) for 48 h. The luciferase activity was quantified using the Dual-Glo Luciferase Assay System (Promega) in an Infinite M Plex microplate reader (Tecan, Crailsheim, Germany).

### 2.12. Statistics

The GraphPad Prism (version 8.43) software was used to perform statistical analysis. All the experiments were performed in at least three independent experiments and are expressed as the means and standard errors of the mean (SEMs). For statistical comparison, analysis of variance (ANOVA) or *t*-tests were used as stated.

## 3. Results

### 3.1. miR-27a-5p and -3p Expression Decreases during Adipogenesis

**Figure 1:**
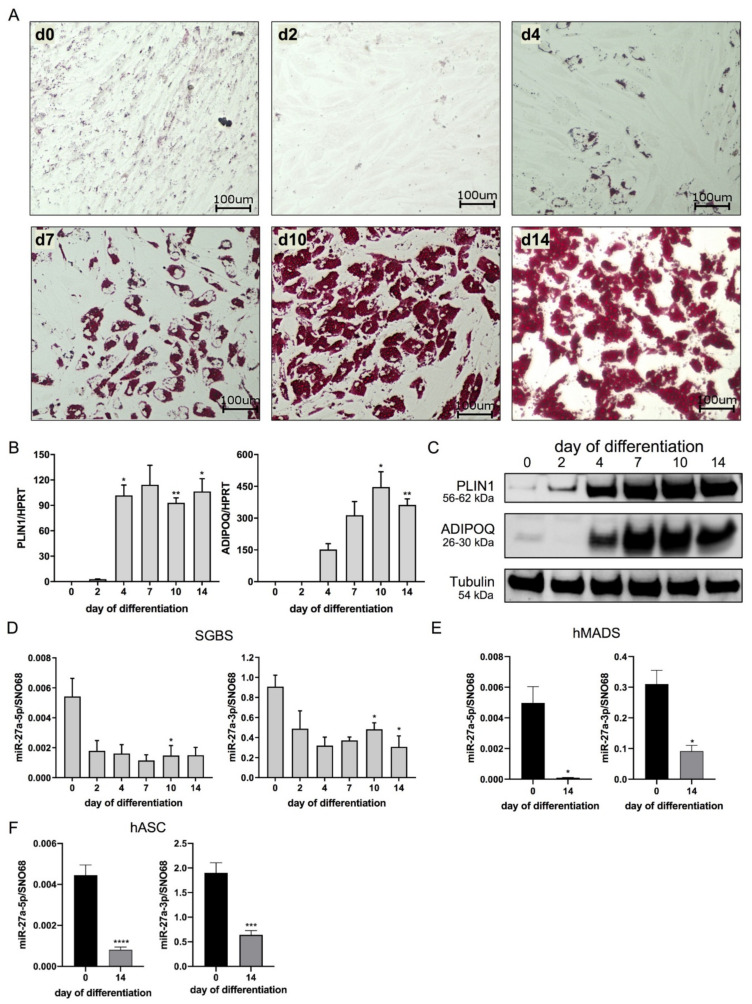
miR-27a-5p and -3p expression decreases during adipogenesis. To assess the expression of miR-27a-5p and -3p during adipogenesis, Simpson–Golabi–Behmel syndrome (SGBS) cells, human multipotent adipose-derived stem cells (hMADS) cells and adipose-derived stromal cells (hASCs) were subjected to adipogenic differentiation. SGBS cells were analyzed on days (d) 0, 2, 4, 7, 10 and 14; hMADS and hASCs, on day 0 and day 14. (**A**) Micrographs of SGBS cells stained with Oil Red O at indicated time points. Lipid droplets are stained in red. (**B**) mRNA expression of adipogenic factors as quantified by qPCR and normalized to HPRT. (**C**) One representative Western blot out of four independent experiments for the adipogenic markers PLIN1 and ADIPOQ with tubulin as loading control. Expression level of miR-27a-5p and -3p in (**D**) SGBS cells, (**E**) hMADS cells and (**F**) hASCs, as quantified by qPCR in relation to SNO68. Statistics: results are displayed as means and SEMs of 4 (**B**,**D**,**E**) and 9 (**F**) independent experiments. One-way ANOVA with Dunnett’s correction (**B**,**D**) and *t*-tests (**E**,**F**) for comparison with day 0; * *p* < 0.05; ** *p* < 0.01; *** *p* < 0.001; **** *p* < 0.0001. SNO68: SNORD68. HPRT: hypoxanthine-guanine phosphoribosyltransferase; PLIN1: perilipin; ADIPOQ: adiponectin.

Human SGBS cells, a non-immortalized cell strain originally isolated from subcutaneous WAT, reflecting key features of human primary adipose-derived stromal cells [21,23], were used as a model system. As depicted in Figure 1 by Oil Red O staining, the cells accumulated lipid droplets during the differentiation process. The differentiation rate after 14 days of adipogenic differentiation was >90%, and cells visibly incorporated increasing amounts of triglycerides over time (Figure 1A, Supplementary Figure S1). In line with this, adipogenic-marker gene expression gradually increased at the mRNA and protein levels (B, C, Supplementary Figure S1B).

miR-27a-5p was, in general, less expressed than miR-27a-3p. During adipogenesis, the expression levels of both miRNAs decreased by 72% for miR-27a-5p and by 66% for miR-27a-3p, when comparing day 0 to day 14 (D). To corroborate the results obtained in SGBS cells, the expression levels in human multipotent adipose-derived stem cells (hMADS) and human primary adipose-derived stromal cells (hASCs) were additionally assessed. In line with the results obtained in SGBS cells, miR-27a-3p was more highly expressed in preadipocytes and in adipocytes than miR-27a-5p. In hMADS cells, the miR-27a-5p and miR-27a-3p levels decreased by ~98% and ~70%, when comparing adipocytes (day 14) with preadipocytes (day 0), and in hASC by ~82% and ~66% (E,F).

### 3.2. miR-27a-3p Regulates Human Adipogenesis

**Figure 2:**
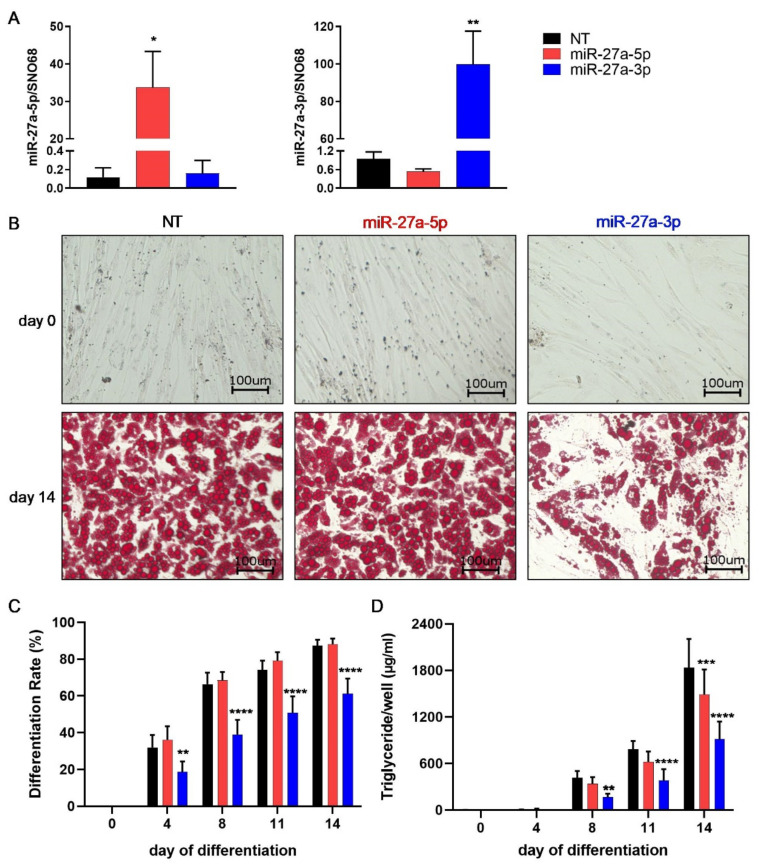
miR-27a-3p decreases adipogenic differentiation rate and triglyceride formation. To assess the effect of miR-27a-5p and -3p on human adipogenesis, SGBS preadipocytes were transfected 48 h prior to adipogenic induction with miRNA mimics or non-target control (NT, 20 nM). (**A**) MiRNA expression on day 0 of adipogenic differentiation (48 h after transfection quantified by qPCR and normalized to SNO68). (**B**) Micrographs of transfected SGBS cells stained with Oil Red O at indicated time points. Lipid droplets are stained in red. (**C**) Differentiation rate and (**D**) triglyceride content on days (d) 0, 4, 8, 11, and 14 during the differentiation process. Statistics: results are displayed as means and SEMs of 5 independent experiments. One-way ANOVA (**A**) and two-way ANOVA (**C**,**D**) with Dunnett’s correction with respect to NT for the same time point; * *p* < 0.05; **p < 0.01; *** *p* < 0.001; **** *p* < 0.0001. SNO68: SNORD68.

To study the impact of miR-27a-5p and -3p on human adipogenesis, miRNA mimics or a non-target control oligonucleotide (NT) were transfected into SGBS preadipocytes 48 h prior to the induction of the differentiation process. At 48 h post-transfection (day 0 of the differentiation protocol), the expression of miR-27a-5p was markedly increased by ~300-fold upon miR-27a-5p mimic transfection compared to NT, while miR-27a-3p expression was unchanged (Figure 2A). Likewise, the transfection of the miR-27a-3p mimic resulted in a ~105 fold elevation, while miR-27a-5p expression was not altered (Figure 2A).

**Figure 3:**
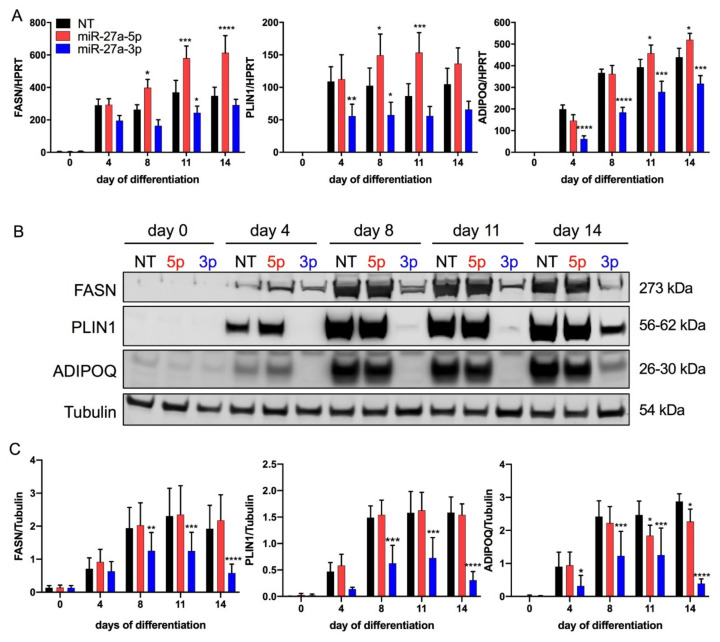
miR-27a-3p-mimic transfection markedly decreases adipogenic-marker expression. To assess the effect of miR-27a-5p and -3p on human adipogenesis, SGBS preadipocytes were transfected 48 h prior to adipogenic induction with miRNA mimics or non-target control (NT, 20 nM). RNA and protein samples were collected on days (d) 0, 4, 8, 11, and 14 of adipogenesis. (**A**) mRNA expression of adipogenic markers as quantified by qPCR normalized to HPRT. (**B**) One representative Western blot out of three independent experiments with fatty-acid synthase (FASN), perilipin (PLIN1) and adiponectin (ADIPOQ), with tubulin as loading control. (**C**) Densitometric analysis of three Western blots. Statistics: results are displayed as means and SEMs of 5 (**A**) and 3 (**C**) independent experiments. Two-way ANOVA with Dunnett’s correction with respect to NT for the same time point; * *p* < 0.05; ** *p* < 0.01; *** *p* < 0.001; **** *p* < 0.0001. HPRT: hypoxanthine-guanine phosphoribosyltransferase. 5p: miR-27a-5p; 3p: miR-27a-3p.

Cell proliferation in the preadipocyte state was unaffected by both miR-27a-5p and -3p transfection (Supplementary Figure S3). For miR-27a-5p, no obvious morphological differences were visible during the adipogenic differentiation process compared to in control cells. In contrast, fewer lipid-laden adipocytes were visible from day 8 to day 14 in miR-27a-3p-mimic-transfected cells (B, Supplementary Figure S2). These observations were in line with a decrease in the differentiation rate on day 14 by 30% and a 50% lower triglyceride content after miR-27a-3p-mimic transfection (C,D). In addition, the adipogenic-marker genes *FASN*, *PLIN1*, and *ADIPOQ* were significantly downregulated after miR-27a-3p transfection at both the mRNA (Figure 3A) and protein levels (B,C). Furthermore, all the effects of miR-27a-3p-mimic transfection on adipogenesis had been proven to be dose dependent (Supplementary Figure S11). miR-27a-5p transfection led to an increase in the mRNA expression of *FASN*, *PLIN1*, and *ADIPOQ*, whereas there was no change at the protein level.

**Figure 4:**
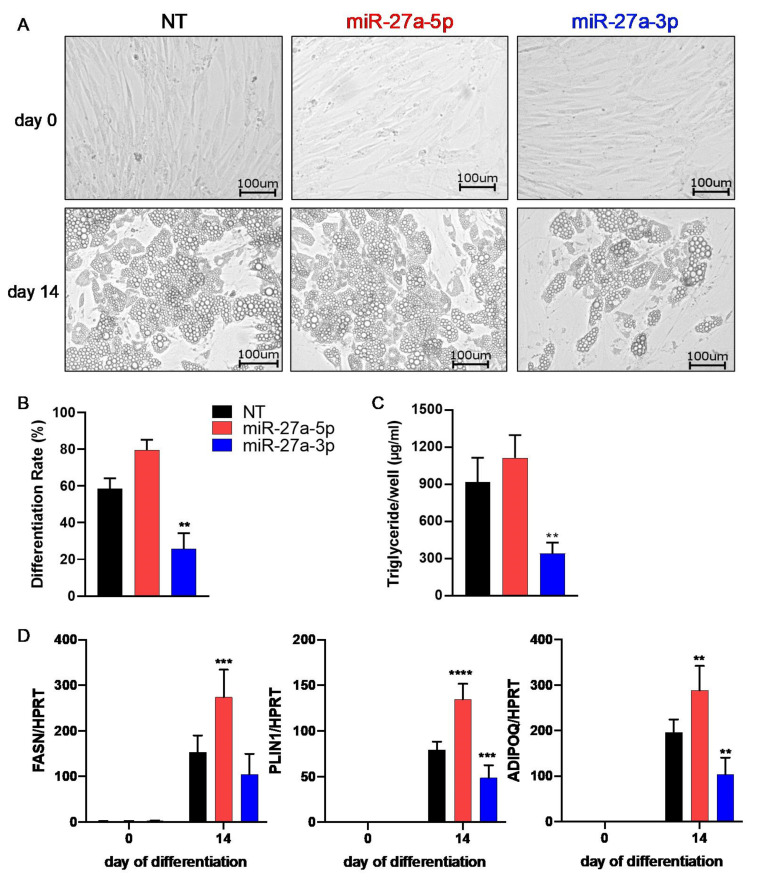
Adipogenic differentiation is inhibited by miR-27a-3p in hMADS cells. To assess the effect of miR-27a-5p and -3p on human multipotent adipose-derived stem cells (hMADS), preadipocytes were transfected 48 h prior to adipogenic induction with miRNA mimics or non-target control (NT, 20 nM). (**A**) Micrographs of transfected hMADS cells at indicated time points. (**B**) Differentiation rate and (**C**) triglyceride content on day 14. (**D**) mRNA expression of adipogenic markers as quantified by qPCR normalized to HPRT. Statistics: results are displayed as means and SEMs of 5 (**B**,**D**) or 3 (**C**) independent experiments. One-way (**B**) and two-way ANOVA (**C**) with Dunnett’s correction with respect to NT of the same time point; ** *p* < 0.01; *** *p* < 0.001; **** *p* < 0.0001. HPRT: hypoxanthine-guanine phosphoribosyltransferase; FASN: fatty-acid synthase; PLIN1: perilipin; ADIPOQ: adiponectin.

We corroborated these findings in a second cell system. Like in SGBS cells, miR-27a-3p-mimic-transfected hMADS cells showed decreased lipid formation (Figure 4A), in line with a lower differentiation rate (B) and lower triglyceride content (C) as well as decreased expression of adipogenic-marker genes (D). Transfection with miR-27a-5p mimics did not affect the adipogenic differentiation rate and triglyceride content, but adipogenic-marker genes such as *FASN*, *PLIN1*, and *ADIPOQ* were significantly increased at the mRNA level.

Taken together, these results demonstrate that miR-27a-3p strongly regulates human adipogenesis. Interestingly miR-27a-3p was upregulated in murine gonadal WAT after 8 weeks of a high-fat diet (HFD) (Supplementary Figure S12), suggesting a potential pathophysiological role of miR-27a-3p in obesity-related WAT dysfunction.

### 3.3. LPL and PPARγ Are Potential miR-27a-3p Targets Involved in Human Adipogenesis

**Figure 5:**
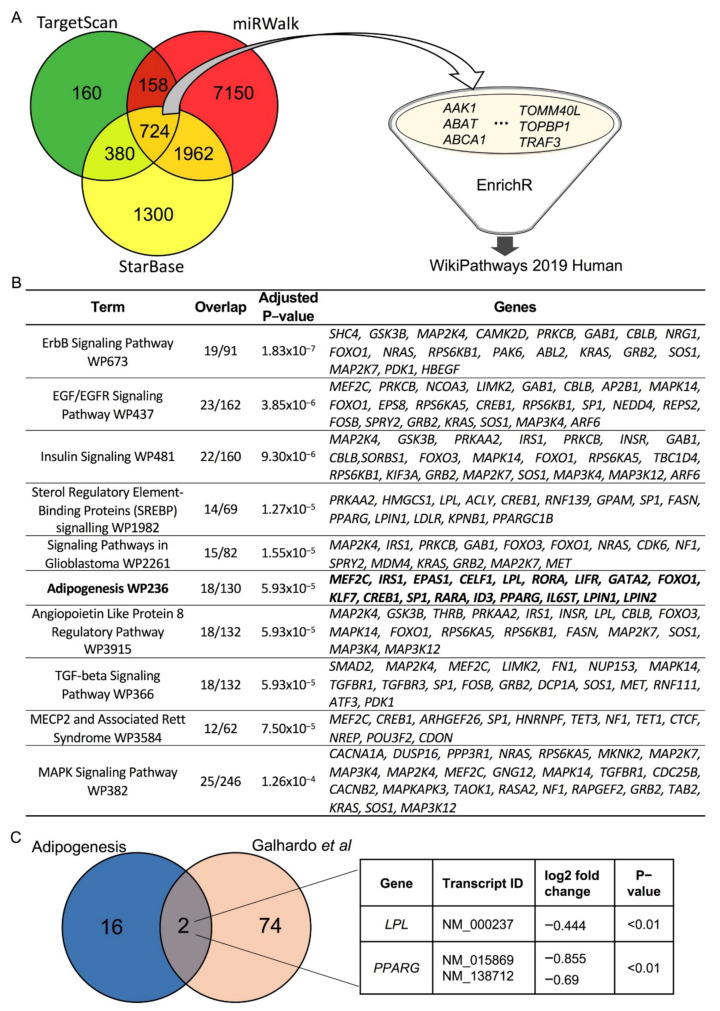
In silico screening for miR-27a-3p targets related to adipogenesis. To identify predicted targets of miR-27a-3p that are involved in adipogenic processes, an intersection analysis with the results of the three databases Targetscan, miRWalk, and Starbase followed by an enrichment analysis using EnrichR was performed [30]. (**A**) Schematic illustration of the in silico analysis. (**B**) Results of the enrichment analysis with EnrichR using WIKIpathway. (**C**) Intersection analysis with genes enriched in the term “Adipogenesis” and target genes of miR-27a shown to be regulated by miR-27a-mimic transfection by Galhardo et al. with log2 fold changes and *p*-values [31]. LPL: lipoprotein lipase; PPARγ: peroxisome proliferator-activated receptor γ.

Next, we aimed to elucidate target genes of miR-27a-3p that would explain the observed phenotype. As miRNAs exert their regulatory function by binding with their seed sequences to their corresponding target mRNAs [33], prediction tools such as TargetScan [29], miRWalk [27], and StarBase [28] can be used to identify possible miRNA–mRNA interactions. Since one miRNA usually targets several genes, these prediction databases contain hundreds to thousands of potential target genes for one single miRNA. To decrease false-positive findings and increase the power of the analysis, we combined an intersection analysis using three prominent prediction algorithms with a subsequent enrichment analysis (Figure 5). A total of 724 target genes were represented in all three databases (A). These were subjected to an enrichment analysis using the online available enrichment tool EnrichR [30] to retrieve predicted target genes that are connected to adipogenic processes. Analyzing this dataset with the biological pathway database WikiPathway revealed 60 significantly enriched pathways. Notably, a pathway termed “Adipogenesis” including 18 predicted target genes for miR-27-3p was within the top significantly enriched categories (adjusted *p*-value < 0.01) (B, Supplementary Table S1). We then compared the in silico data with transcriptomic data available from a study published by Galhardo et al. [31]. In their study, transcriptomic analysis was performed in SGBS cells differentiated for 4 days and then transfected for 24 h with a miR-27a mimic in order to identify metabolism-related transcription factors [31]. They found 76 genes being downregulated (*p* < 0.01) and that contain a binding site for miR-27a in their 3′ UTRs. An additional intersection analysis of these 76 genes crossed with our in silico data identified lipoprotein lipase (LPL) and peroxisome proliferator-activated receptor γ (PPARγ) as potential miR-27a-3p targets (Figure 5C). Next, we aimed to verify whether these target genes were indeed downregulated in our in vitro system.

### 3.4. LPL Is Directly Regulated by miR-27a-3p but Does Not Influence Human Adipogenesis

**Figure 6:**
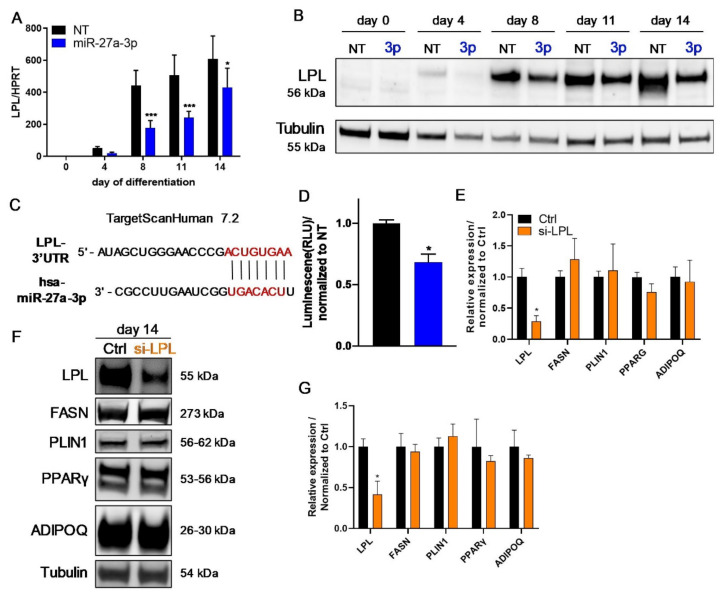
The miR-27a-3p target LPL does not alter adipogenesis. To assess if miR-27a-3p regulated its predicted target, lipoprotein lipase (LPL), SGBS preadipocytes were transfected 48 h prior to adipogenic induction with miRNA mimics or non-target control (NT, 20 nM), and additionally, a dual-luciferase reporter assay was performed in HEK293 cells. (**A**) mRNA level of LPL as quantified by qPCR normalized to HPRT at indicated time points of adipogenic differentiation. (**B**) One representative Western blot from three independently performed experiments for LPL using tubulin as loading control. (**C**) Illustration of the predicted binding site for miR-27a-3p in the 3′ UTR in the human *LPL* mRNA. (**D**) Results of the dual-luciferase reporter assay performed in HEK293 cells. Luminescence from firefly/Renilla luciferase activity was determined as indicated. (**E**–**G**) SGBS preadipocytes were transfected 48 h prior to adipogenic induction with either control non-target (Ctrl) or an siRNA pool targeting human LPL (si-LPL, 20 nM). RNA and protein samples were collected on day 14 of the differentiation process. (**E**) mRNA expression of LPL and adipogenic markers as quantified by qPCR normalized to HPRT after LPL knockdown. (**F**) One representative Western blot out of three independent experiments after LPL knockdown in SGBS cells. (**G**) Densitometric analysis of all Western blots on day 14 of adipogenesis normalized to tubulin. Statistics: results are displayed as means and SEMs of 5 (**A**) and 3 (**B**,**D**–**G**) independent experiments. Two-way ANOVA with Bonferroni correction (**A**); *t*-tests (**D**,**E**,**G**) with respect to NT/Ctrl for the same time point; * *p* < 0.05; *** *p* < 0.001. HPRT: hypoxanthine-guanine phosphoribosyltransferase; FASN: fatty-acid synthase; PLIN1: perilipin; PPARγ: peroxisome proliferator-activated receptor γ; ADIPOQ: adiponectin; 3p: miR-27a-3p.

The transfection of SGBS preadipocytes with miR-27a-3p mimic resulted in a strong decrease in LPL mRNA and protein levels during the process of adipogenesis (Figure 6A,B, Supplementary Figure S4). In line with this, miR-27a-3p-mimic transfection resulted in a 54% decrease in *LPL* mRNA expression in hMADS cells on day 14 (Supplementary Figure S5). To verify the direct regulation of *LPL* by miR-27a-3p, a reporter assay using the pmiRGLO vector with the annotated binding site for miR-27a-3p in the 3′ UTR of *LPL* was performed (C). As shown in Figure 6D, miR-27a-3p-mimic co-transfection led to a significant decrease in the relative luminescence compared to NT, indicating that the miRNA indeed interacts with its binding site in the *LPL* gene.

To assess if the miR-27a-3p target *LPL* regulates human adipogenesis, we performed siRNA-mediated knockdown experiments. The transfection of 20 nM siRNA targeting LPL led to a ~72% decrease in *LPL* mRNA and ~55% decrease in protein abundance compared to control transfections (Ctrl) (Figure 6E–G; Supplementary Figure S7B). The knockdown of LPL had no impact on the morphology of SGBS cells during the adipogenic differentiation process compared to Ctrl (Supplementary Figure S6), nor did we observe any difference in the differentiation rate or triglyceride content (Supplementary Figure S7A). Likewise, adipogenic-marker gene expression was unchanged at the mRNA as well as protein level (Figure 6E–G; Supplementary Figure S7C–F). Therefore, we confirm that *LPL* is a direct target gene of miR-27a-3p but is not responsible for its inhibitory effect on adipogenesis.

### 3.5. The Transcription Factor PPARγ Is Directly Regulated by miR-27a-3p

**Figure 7:**
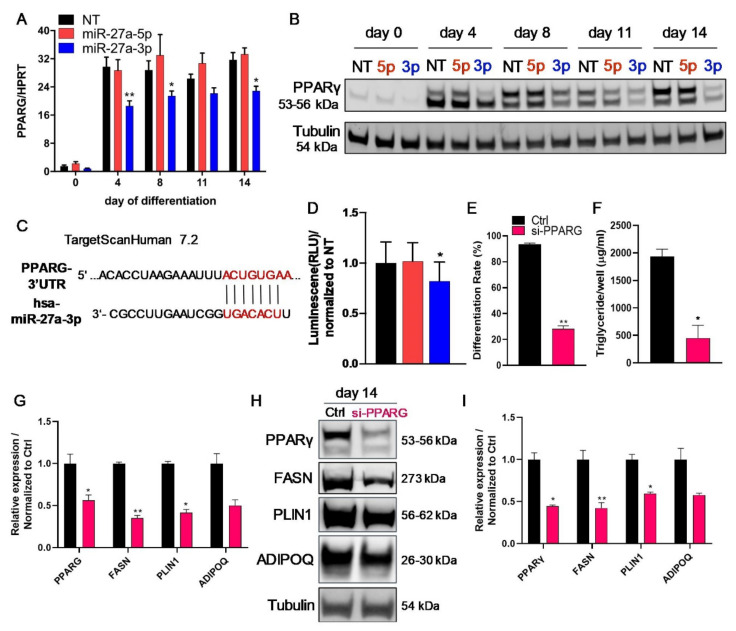
PPARγ is a direct target of miR-27a-3p regulating adipogenesis. To assess if miR-27a-3p regulated its predicted target PPARγ, SGBS preadipocytes were transfected 48 h prior to adipogenic induction with miRNA mimics or non-target control (NT, 20 nM), and additionally, a dual-luciferase reporter assay was performed in HEK293 cells. (**A**) mRNA level of PPARG as quantified by qPCR normalized to HPRT at indicated timepoints of adipogenic differentiation. (**B**) One representative Western blot from three independently performed experiments for PPARγ using tubulin as loading control. (**C**) Illustration of the predicted binding site for miR-27a-3p in the 3′ UTR in the human *PPARG* mRNA. (**D**) Results of the dual-luciferase reporter assay performed in HEK293 cells. Luminescence from firefly/Renilla luciferase activity was determined as indicated. (**E**–**I**) SGBS preadipocytes were transfected 48 h prior to adipogenic induction with either control non-target (Ctrl) or an siRNA pool targeting human PPARG (si-PPARG, 20 nM). Triglycerides, and RNA and protein samples were collected on day 14 of the differentiation process. (**E**) Differentiation rate and (**F**) triglyceride content were measured on day 14. (**G**) mRNA expression of PPARG and adipogenic markers was quantified by qPCR, normalized to HPRT, after PPARG knockdown. (**H**) One representative Western blot out of three independent experiments after PPARG knockdown in SGBS cells. (**I**) Densitometric analysis of all Western blots was performed on day 14 of adipogenesis, normalized to tubulin. Statistics: results are displayed as means and SEMs of 5 (**A**) and 3 (**D**–**G**,**I**) independent experiments. Two-way ANOVA (**A**) and one-way ANOVA (**D**) with Dunnett’s correction with respect to NT for the same time point; *t*-test (**E**–**G**,**I**) with respect to Ctrl ; * *p* < 0.05; ** *p* < 0.01. HPRT: hypoxanthine-guanine phosphoribosyltransferase; PPARG/PPARγ: peroxisome proliferator-activated receptor γ; FASN: fatty-acid synthase; PLIN1: perilipin; ADIPOQ: adiponectin; 5p: miR-27a-5p; 3p: miR-27a-3p.

To assess if the second predicted miR-27a-3p target, PPARγ, is regulated by miR-27a-3p, we first analyzed its mRNA expression and protein level during adipogenesis after miR-27a-3p-mimic delivery in SGBS preadipocytes. As depicted in Figure 7A, miR-27a-3p mimic transfection resulted in a significant downregulation of *PPARG* mRNA expression at the studied timepoints of adipogenesis. At the protein level, this effect was even more prominent, displaying a reduction of ~82% on day 14 (Figure 7B; Supplementary Figure S8A), suggesting that both mRNA silencing and cleavage take place as repressional mechanisms. In line, miR-27a-3p inhibition led to a significant increase in PPARγ protein expression, and increased triglyceride incorporation and adipogenic-marker gene expression (Supplementary Figure S10). In contrast to this, miR-27a-5p had no effect on *PPARG* mRNA or protein expression. These findings were also confirmed in hMADS cells (Supplementary Figure S8B). We demonstrated a direct regulation of PPARγ by miR-27a-3p using a pmiRGLO dual-luciferase reporter assay (Figure 7C,D). We confirmed the important role of PPARγ for human adipogenesis by knocking down PPARγ using siRNA (Figure 7E–I; Supplementary Figure S9). Efficient knockdown was verified at the mRNA and protein levels (Figure 7G–I). A knockdown of PPARγ by 55% at the protein level resulted in a reduction in the adipogenic differentiation rate by 70% and a decrease in incorporated triglycerides by 77% (Figure 7E,F). In addition, the adipogenic-marker genes *FASN* and *PLIN1* were significantly reduced (Figure 7G–I).

## 4. Discussion

Over the last few years, miRNAs have gained considerable attention in the field of metabolism, obesity, and related disorders [10,34]. Prominent examples are miR-103 and miR-107, which were among the first miRNAs described to play important roles in the regulation of insulin sensitivity, and miR-365, which is a key regulator of insulin secretion in pancreatic islets [35,36]. miR-27a was recently discovered as a crucial regulator of insulin sensitivity in skeletal muscle and murine adipocytes and might, thereby, be an important fine tuner of systemic glucose metabolism [37,38]. Interestingly, Yu et al. showed that murine adipocyte-derived miR-27a acts as a negative regulator of insulin sensitivity in skeletal muscle cells [37], underlining the importance of adipose tissue as a rich source of miRNAs with systemic effects [39]. Based on these findings, we were interested in the regulatory function of miR-27a in human adipocytes. As recent studies confirmed that, especially under pathological conditions, not only the guide strand of a miRNA but also the passenger strand is able to exert important functions [19,40,41], we performed, for the first time, a systematic study on the role of miR-27a-3p and -5p in human adipocytes.

We found that miR-27a-3p and -5p strongly decreased during adipogenic differentiation in three human in vitro model systems, as had already been shown in other adipogenic in vitro model systems [42,43,44,45,46,47,48].

The new formation of adipocytes is an important process for regulating fat mass, and certain metabolic disorders are associated with defects in adipocyte differentiation and lipid metabolism [49,50,51,52], where miRNAs play a crucial role in maintaining adipocyte homeostasis [34]. A number of miRNA species are known to regulate adipogenesis, among them being miR-27a/b, which was shown to inhibit adipogenesis in 3T3-L1 cells [45]. These findings were also confirmed by others not only in murine [44,46,47,53,54] but also in ovine, bovine, and porcine model systems [48,55,56]. We therefore hypothesized that miR-27a might be a potential regulator of human adipogenesis as well. In line with recent publications, we show that miR-27a-3p represses human adipogenesis [42,43,44], while we are the first to show that miR-27a-5p had no negative effect on adipocyte formation. Interestingly, miR-27a-5p mimic transfection even led to an increase in adipogenic markers such as *FASN, PLIN1*, and *ADIPOQ* at the mRNA level in SGBS as well as in hMADS cells. However, the differentiation rates, triglyceride formation, and adipocyte-marker protein expression were not significantly altered by miR-27a-5p. Both model systems used in our study are characterized by a very high potential for adipogenic differentiation. They are therefore very likely to detect an inhibition of adipogenesis, but additional experimental setups would be required to prove a stimulatory effect of miR-27a-5p. Though miR-27a-5p had no significant effect on human-adipocyte differentiation, it seems to be an important modulator of NF-kB signaling in human aortic endothelial cells [57] and a regulator of liver fat deposition in the bovine liver [58], showing that passenger strands of miRNAs also exert relevant regulatory functions. Other studies found that miR-27a promotes cell proliferation in ovine preadipocytes [48] and hepatocellular carcinoma cells [59] and an anti-proliferative effect of miR-27a-5p in prostate cancer cells [60]. However, we did not observe any obvious difference in preadipocyte proliferation and cell viability between control and miR-27a-5p-/-3p-mimic transfected cells. Taken together, we describe, in our study, that only miR-27a-3p, but not miR-27a-5p, has an inhibitory effect on human-adipocyte differentiation.

MiR-27a-3p was significantly increased in murine WAT after a HFD, underlining the important physiological role of this miRNA in adipose tissue. This finding is in line with previous reports and suggests a potential role of miR-27a-3p as a mediator of obesity-related adipocyte dysfunction [38,45]. Lin et al. report a hypoxia-related upregulation of miR-27a, and Chen et al., an inflammation-driven increase in miR-27a in WAT [38,45]. This is in line with our initial report that miR-27a is upregulated in an in vitro model system for WAT inflammation [20].

Using an in silico screening approach combined with published transcriptome data [31], we identified target genes of miR-27a-3p that explain the decrease in adipogenic potential. Here, *LPL* and *PPARG* were predicted as potential targets of miR-27a-3p.

*LPL* expression was decreased upon mimic transfection in SGBS and hMADS cells, and a luciferase-based miRNA-target-site reporter assay verified the direct regulatory function of miR-27a-3p on *LPL*. LPL is a member of the lipase gene family and catalyzes the cleavage (hydrolysis) of triacylglycerols from lipoproteins, such as those found in chylomicrons and very-low-density lipoproteins (VLDL), and is expressed in adipocytes, myocytes, and macrophages [61]. A functional deficiency of LPL leads to human familial chylomicronemia and ectopic lipid deposition [61]. *LPL*-knockout mice exhibit very high serum triglyceride levels directly after birth and die early from a lethal engorgement of pulmonary capillaries with chylomicrons [62]. Here, we examined the role of LPL in human adipogenesis. Whereas previous studies showed a clear regulatory function of LPL, we did not observe any difference in this regard. Neither adipogenic-marker gene expression nor triglyceride formation were altered upon siRNA-mediated LPL knockdown. It was shown earlier that the inhibition of LPL in 3T3-L1 adipocytes caused a reduction in lipid accumulation [63], and a knockdown of LPL in hASCs reduced their adipogenic potential, while LPL overexpression had pro-adipogenic effects [64]. However, in contrast to this, the knockdown of LPL in bovine adipocytes increased lipid storage [65]. In our study, *LPL* expression was clearly regulated by miR-27a-3p but did not alter the adipogenic differentiation process. We want to emphasize that the differentiation rates appeared to be very low in the corresponding microphotographs of both studies claiming a pro-adipogenic effect of LPL [63,64], while our model system is characterized by very high rates of adipogenic differentiation. This might explain the contradictory results.

The second potential target gene we identified was *PPARG*, a transcription factor and essential regulator of adipogenesis, energy balance, and lipid metabolism [66,67]. PPARγ protein expression was strongly reduced upon miR-27a-3p-mimic transfection, and we further proved the direct binding of miR-27a-3p to the 3′ UTR of *PPARG* using a luciferase-based miRNA reporter assay. This is in line with several publications using murine [38,46,68,69], rat [53], porcine [56], sheep [48], and bovine [55] model systems. Additionally, miR-27b, which differs in only one base pair from miR-27a [70], also targets PPARγ and regulates differentiation in hMADS cells [71] and rat chondrocytes [72]. In addition, Kulyté et al. described a regulation of PPARγ by miR-27a/b-3p in human ASCs [43]. The strong inhibition of PPARγ expression by miR-27a plausibly explains the impact of miR-27a-3p on adipogenesis, as a knockdown of PPARγ strongly reduced the adipogenic differentiation capacity. This is in line with previous reports that PPARγ is essential for the formation of adipocytes in vitro and in vivo [43,73].

## 5. Conclusions

Taken together, both miR-27a-5p and -3p are downregulated during human adipogenesis and miR-27a-3p reduces adipogenesis by directly targeting *PPARG*. Interestingly, this process seems to be exclusive for miR-27a-3p, while miR-27a-5p has no effect on the adipogenic potential of human preadipocytes. Though we confirmed *LPL* as an additional target of miR-27a-3p, it does not appear to be involved in regulating human adipogenesis, as was previously reported in other studies. Nevertheless, targeting LPL with miR-27a-3p might have important implications in diseases associated with decreased LPL function, such as hypertriglyceridemia.

## Data Availability

All the data relevant to the present study are available on request from the corresponding author.

## Supplementary Materials

The following are available online at https://www.mdpi.com/article/10.3390/cells10113205/s1, Figure S1: Verification of SGBS cell differentiation, Figure S2: Micrographs of miR-27a-5p and -3p gain of function in SGBS cells, Figure S3: miR-27a-5p and -3p do not affect cell proliferation in SGBS preadipocytes, Figure S4: Densitometric analysis of LPL in SGBS cells transfected with miR-27a-3p, Figure S5: miR-27a-3p decreases LPL expression in hMADS cells, Figure S6: Knockdown of LPL does not alter morphology of SGBS cell differentiation, Figure S7: Adipogenic differentiation is not affected by LPL knockdown, Figure S8: PPARγ is regulated by miR-27a-3p in hMADS cells, Figure S9: Knock-down of PPARγ impairs adipogenesis of SGBS cells, Figure S10: Adipogenic differentiation is enhanced by miR-27a-3p inhibitor in SGBS cells, Figure S11: Lower dose of miR-27a-3p can decrease adipogenic differentiation in SGBS cells, Figure S12: miR-27a-3p and -5p expression is increased in gonadal WAT after 8 weeks of high-fat diet (HFD), Table S1: Significant results of the EnrichR analysis for WikiPathway.
