# Supplementary material for: microRNA-27a-3p but Not -5p Is a Crucial Mediator of Human Adipogenesis
Source: Cells. 2021 Nov 17;10(11):3205. doi: 10.3390/cells10113205 (PMC8625276; doi:10.3390/cells10113205)
Supplement: Supplementary file 1 [file cells-10-03205-s001.zip › cells-1459249 - Supp Mat NEW.pdf]

Supplementary Materials

microRNA-27a-3p but Not -5p Is a Crucial Mediator of Human Adipogenesis

Hang Wu [1], Taner Pula [1], Daniel Tews [2], Ez-Zoubir Amri [3], Klaus-Michael Debatin [1], Martin Wabitsch [2], Pamela Fischer-Posovszky [1] and Julian Roos [1,]*

[1] Department of Pediatrics and Adolescent Medicine, Ulm University Medical Center, 89075 Ulm, Germany; hang.wu@uni-ulm.de (H.W.); taner.pula@uni-ulm.de (T.P.); klaus-michael.debatin@uniklinik-ulm.de (K.-M.D.); Pamela.Fischer@uniklinik-ulm.de (P.F-P..)

[2] Division of Pediatric Endocrinology and Diabetes, Department of Pediatrics and Adolescent Medicine, Ulm University Medical Center, 89075 Ulm, Germany; Daniel.Tews@uniklinik-ulm.de (D.T.); martin.wabitsch@uniklinik-ulm.de (M.W.)

[3] Inserm, CNRS, iBV, Université Côte d'Azur, 06103 Nice, France; Ez-Zoubir.Amri@unice.fr (E-Z.A)

* Correspondence: julian.roos@uni-ulm.de; Tel.: +49 731 500 57255

Citation: Wu, H.; Pula, T.; Tews, D.; Amri, E.-Z.; Debatin, K.-M.; Wabitsch, M.; Fischer-Posovszky, P.; Roos, J. microRNA-27a-3p but Not -5p Is a Crucial Mediator of Human Adipogenesis. *Cells* **2021**, *10*, x. https://doi.org/10.3390/cells10113205

Academic Editor: Ajit Vikram

Received: 27 October 2021
Accepted: 11 November 2021
Published: 17 November 2021

Publisher's Note: MDPI stays neutral with regard to jurisdictional claims in published maps and institutional affiliations.

Copyright: © 2021 by the authors. Licensee MDPI, Basel, Switzerland. This article is an open access article distributed under the terms and conditions of the Creative Commons Attribution (CC BY) license (https://creativecommons.org/licenses/by/4.0/).

Supplementary Materials:

Figure S1: Verification of SGBS cell differentiation.

Figure S2: Micrographs of miR-27a-5p and -3p gain-of-function in SGBS cells.

Figure S3: MiR-27a-5p and -3p do not affect cell proliferation in SGBS pre-adipocytes.

Figure S4: Densitometric analysis of LPL in SGBS cell transfected with miR-27a-3p.

Figure S5: MiR-27a-3p decreases LPL expression in hMADS cells.

Figure S6: Knockdown of LPL does not alter morphology of SGBS cell differentiation.

Figure S7: Adipogenic differentiation is not affected by LPL knockdown.

Figure S8: PPARγ is regulated by miR-27a-3p in SGBS and hMADS cells.

Figure S9: Knock-down of PPARγ impairs adipogenesis of SGBS cells.

Figure S10: Adipogenic differentiation is enhanced by miR-27a-3p inhibitor in SGBS cells.

Figure S11: Lower dose of miR-27a-3p can decrease adipogenic differentiation in SGBS cells.

Figure S12: miR-27a-3p and -5p expression is increased in gonadal WAT after 8 weeks of high-fat diet (HFD).

Table S1: Significant results of the EnrichR analysis for WikiPathway.

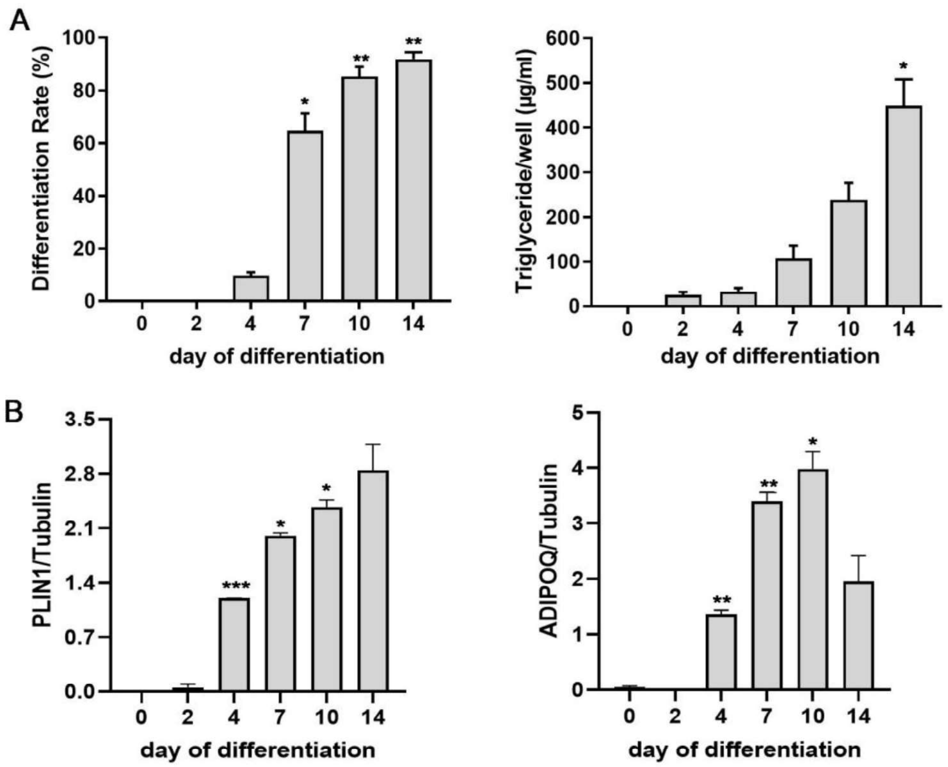

Figure S1. Verification of SGBS cell differentiation. (A) Differentiation rate and triglyceride content on day 0, 2, 4, 7, 10 and 14 during SGBS cell differentiation process. (B) Densitometric analysis of Western blot experiments during SGBS adipogenesis. Statistics: results are displayed as mean +SEM of four independent experiments. One-way ANOVA with Dunnett correction related to day 0; *p<0.05; **p<0.01; ***p<0.001. PLIN1: perilipin, ADIPOQ: adiponectin.

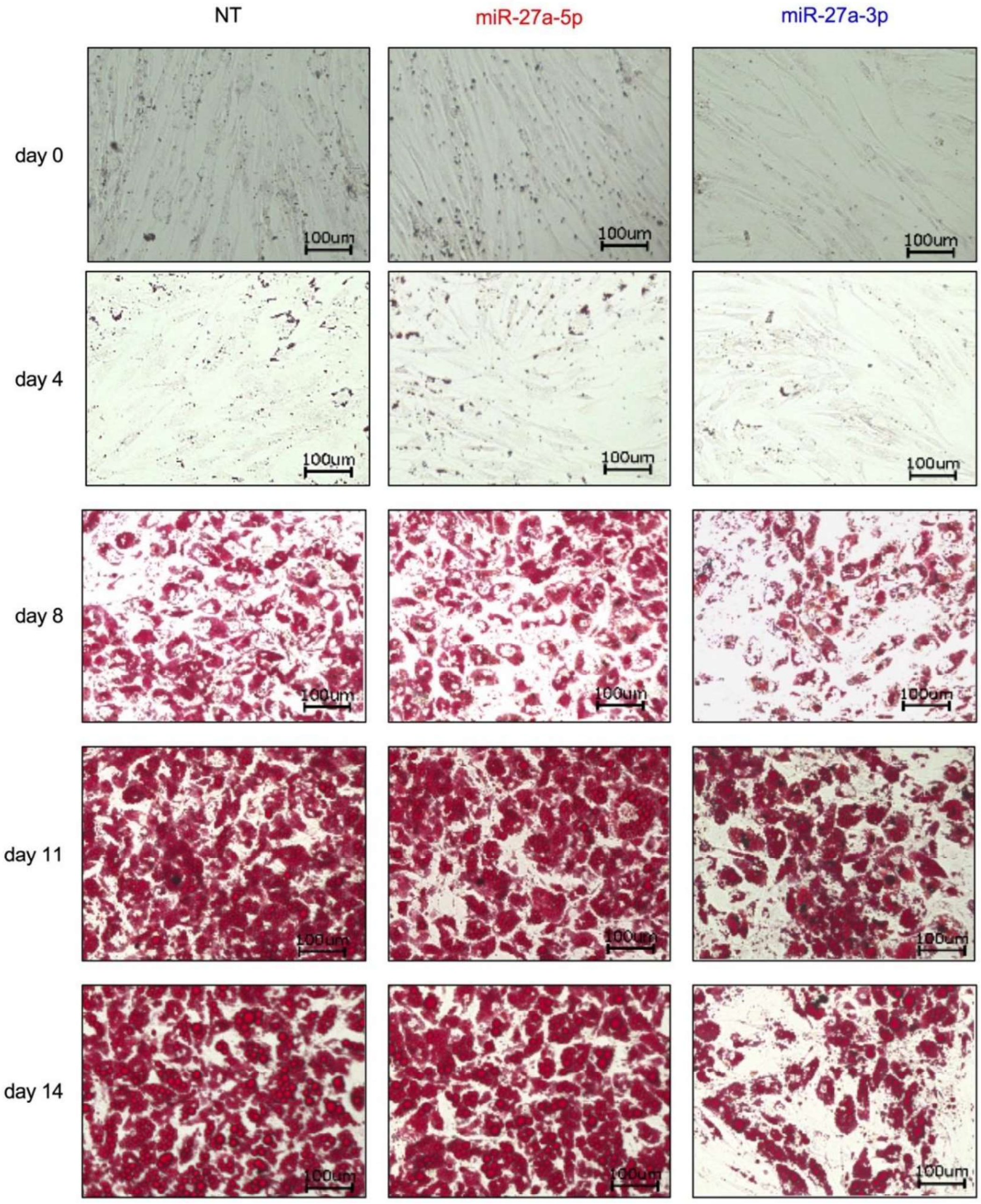

Figure S2. Micrographs of miR-27a-5p and -3p gain-of-function in SGBS cells. To assess the effect of miR-27a-5p and -3p on human adipogenesis, SGBS preadipocytes were transfected 48h prior adipogenic induction with miRNA mimics or non-target control (NT, 20nM). Cells were stained with Oil red O at indicated time points. Lipid droplets are stained in red.

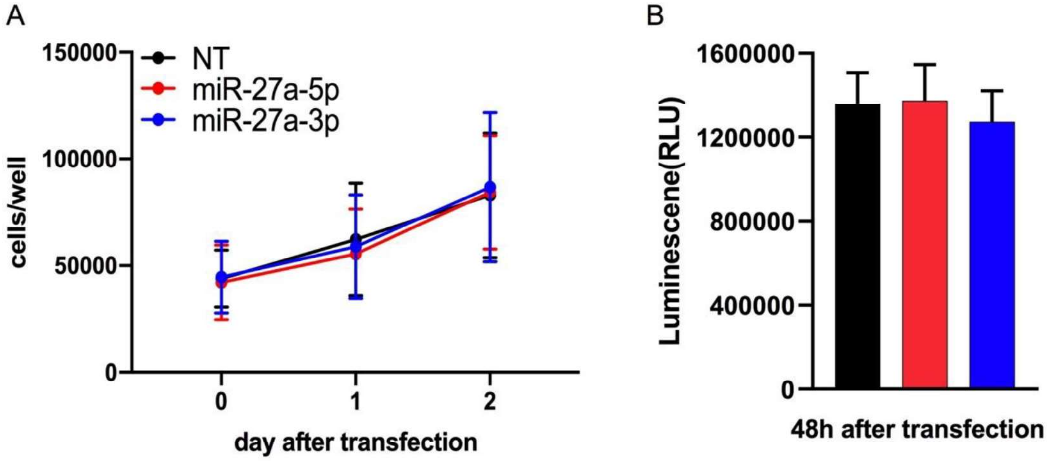

Figure S3. miR-27a-5p and -3p do not affect cell proliferation in SGBS pre-adipocytes. To assess the effect of miR-27a-5p and -3p on SGBS cell proliferation, SGBS preadipocytes were transfected with miRNA mimics or non-target control (NT, 20nM) 48h prior adipogenic induction. (A) SGBS preadipocytes were counted just before transfection and 24h, 48h post transfection. (B) CellTiter-Glo assay was applied 48h post transfection among all three conditions (NT/miR-27a-5p/-3p). Statistics: results are displayed as mean and SEM of three independent experiments. One-way ANOVA with Dunnett correction related to NT of the same time point.

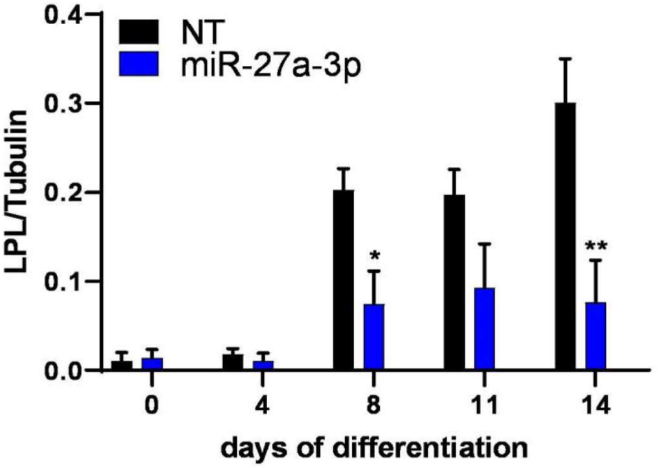

Figure S4. Densitometric analysis of LPL in SGBS cell transfected with miR-27a-3p. To assess if miR-27a-3p regulates its predicted target Lipoprotein Lipase (LPL), SGBS preadipocytes were transfected with miRNA mimics or non-target control (NT, 20nM) 48h prior adipogenic induction. Protein samples were collected on day 0, 4, 8, 11, 14 of adipogenesis. Densitometric analysis of three Western blots of LPL with Tubulin as loading control. Statistics: results are displayed as mean and SEM of three independent experiments. Two-way ANOVA with Bonferroni correction related to NT of the same time point; *p<0.05; **p<0.01.

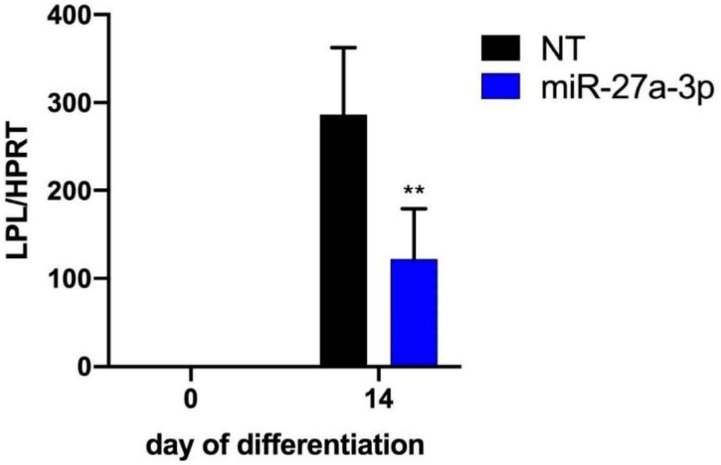

Figure S5. miR-27a-3p decreases LPL expression in hMADS cells. To assess if miR-27a-3p regulates its predicted target Lipoprotein Lipase (LPL), hMADS preadipocytes were transfected with miRNA mimics or non-target control (NT, 20nM) 48h prior adipogenic induction. RNA samples were collected on day 0 and 14 of adipogenesis. LPL mRNA expression quantified by qPCR related to HPRT. Statistics: results are displayed as mean +SEM of five independent experiments. Two-way ANOVA with Dunnett correction related to NT of the same time point; *p<0.05. HPRT: Hypoxanthine-Guanin-Phosphoribosyltransferase. hMADS: human multipotent adipose-derived stem cells.

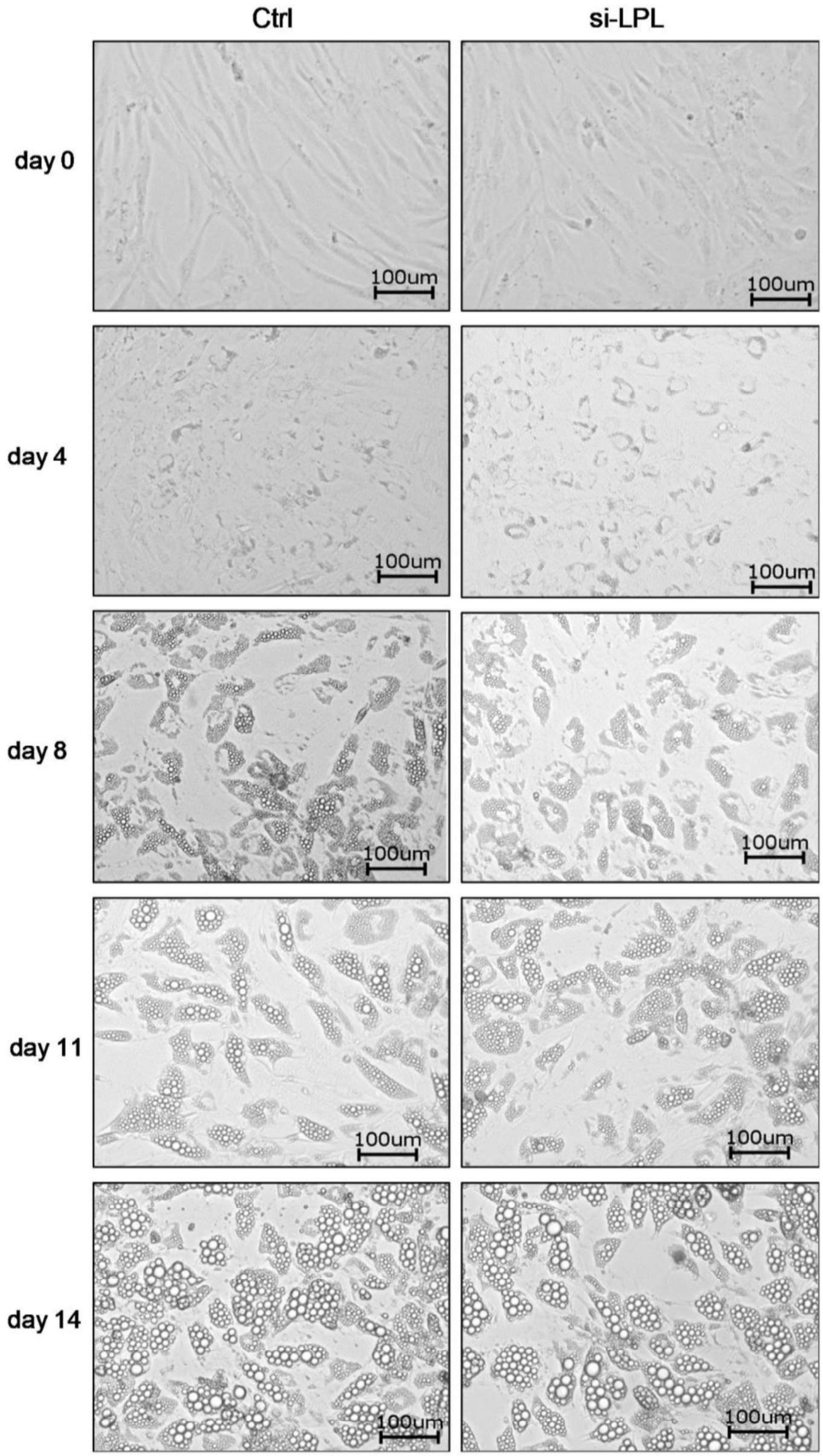

Figure S6. Knockdown of LPL does not alter morphology of SGBS cell differentiation. To assess if the miR-27a-3p target LPL regulates human adipogenesis, SGBS preadipocytes were transfected 48h prior adipogenic induction with either control non-target (Ctrl) or an siRNA pool targeting human LPL (si-LPL, 20nM). Micrographs were taken at indicated time points.

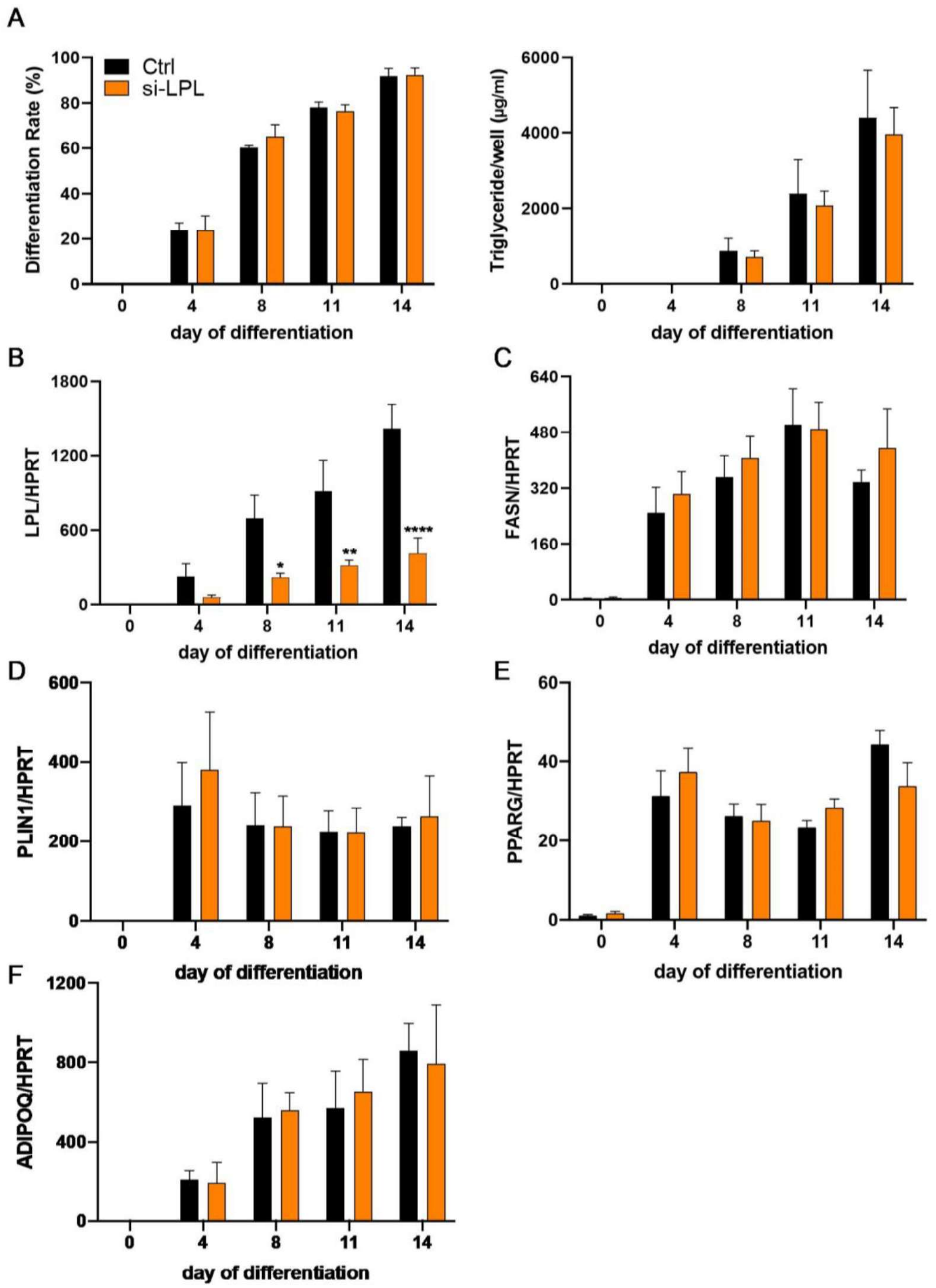

Figure S7. Adipogenic differentiation is not affected by LPL knockdown. To assess if the miR-27a-3p target LPL regulates human adipogenesis, SGBS preadipocytes were transfected with either control non-target (Ctrl) or an siRNA pool targeting human LPL (si-LPL, 20nM) 48h prior adipogenic induction. (A) Differentiation rate and triglyceride content at indicated time points during SGBS cell differentiation. mRNA expression of LPL (B) and adipogenic markers (C-F) quantified by qPCR related to HPRT. Statistics: results are displayed as mean +SEM of three independent experiments. Two-way ANOVA with Bonferroni correction related to Ctrl of the same time point; *$p<0.05$; **$p<0.01$; ****$p<0.0001$. HPRT: Hypoxanthine-Guanin-Phosphoribosyltransferase, LPL: lipoprotein lipase, FASN: fatty acid synthase, PLIN1: perilipin, PPARγ: peroxisome proliferator-activated receptors γ, ADIPOQ: adiponectin.

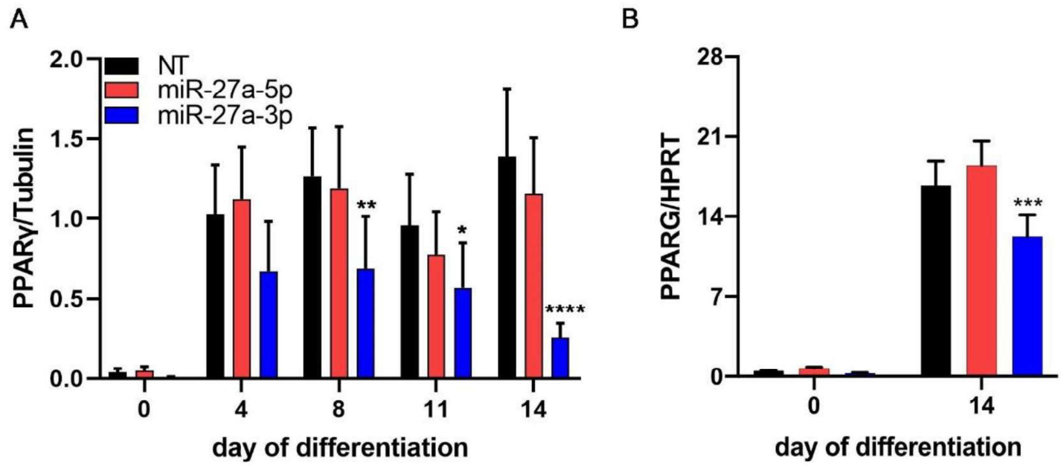

Figure S8. PPARγ is regulated by miR-27a-3p in SGBS and hMADS cells. To assess if miR-27a-3p regulates its predicted target PPARγ, SGBS or hMADS preadipocytes were transfected with miRNA mimics or non-target control (NT, 20nM) 48h prior adipogenic induction. (A) Densitometric analysis of three Western blots of PPARγ with Tubulin as loading control in SGBS cells. (B) mRNA expression of PPARG was quantified by qPCR related to HPRT in hMADS cells. Statistics: results are displayed as mean + SEM of 3 independent experiments. Two-way ANOVA with Dunnett correction related to NT of the same time point; *p<0.05, **p<0.01, ***p<0.001, ****p<0.0001. HPRT: Hypoxanthine-Guanin-Phosphoribosyltransferase, PPARγ/PPARG: Peroxisome Proliferator-activated Receptor γ.

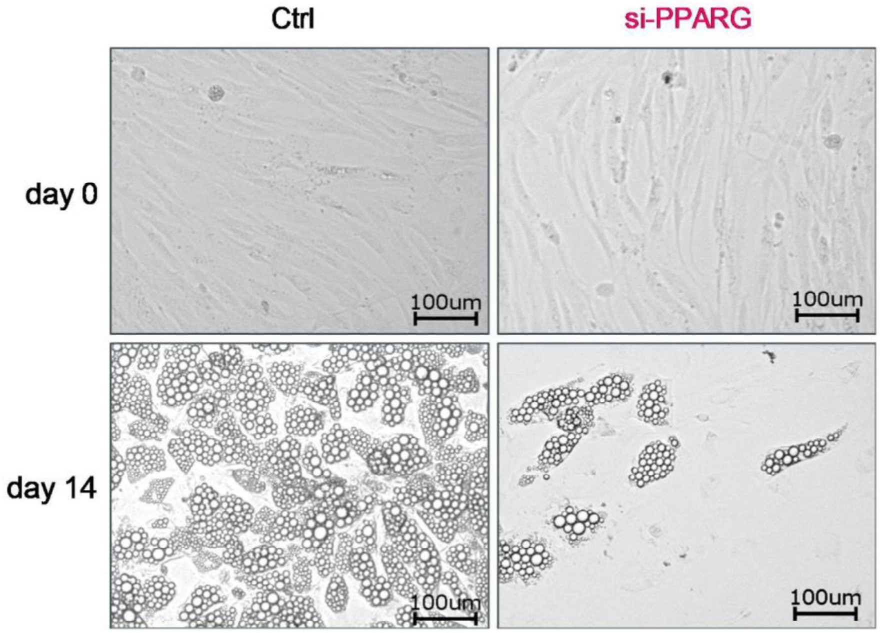

Figure S9. Knock-down of PPARγ impairs adipogenesis of SGBS cells. To assess if the miR-27a-3p target PPARγ regulates human adipogenesis, SGBS preadipocytes were transfected 48h prior adipogenic induction with either control non-target (Ctrl) or an siRNA pool targeting human PPARG (si-PPARG, 20nM). Microphotographs were taken at indicated time points. PPARG: Peroxisome Proliferator-activated Receptor γ.

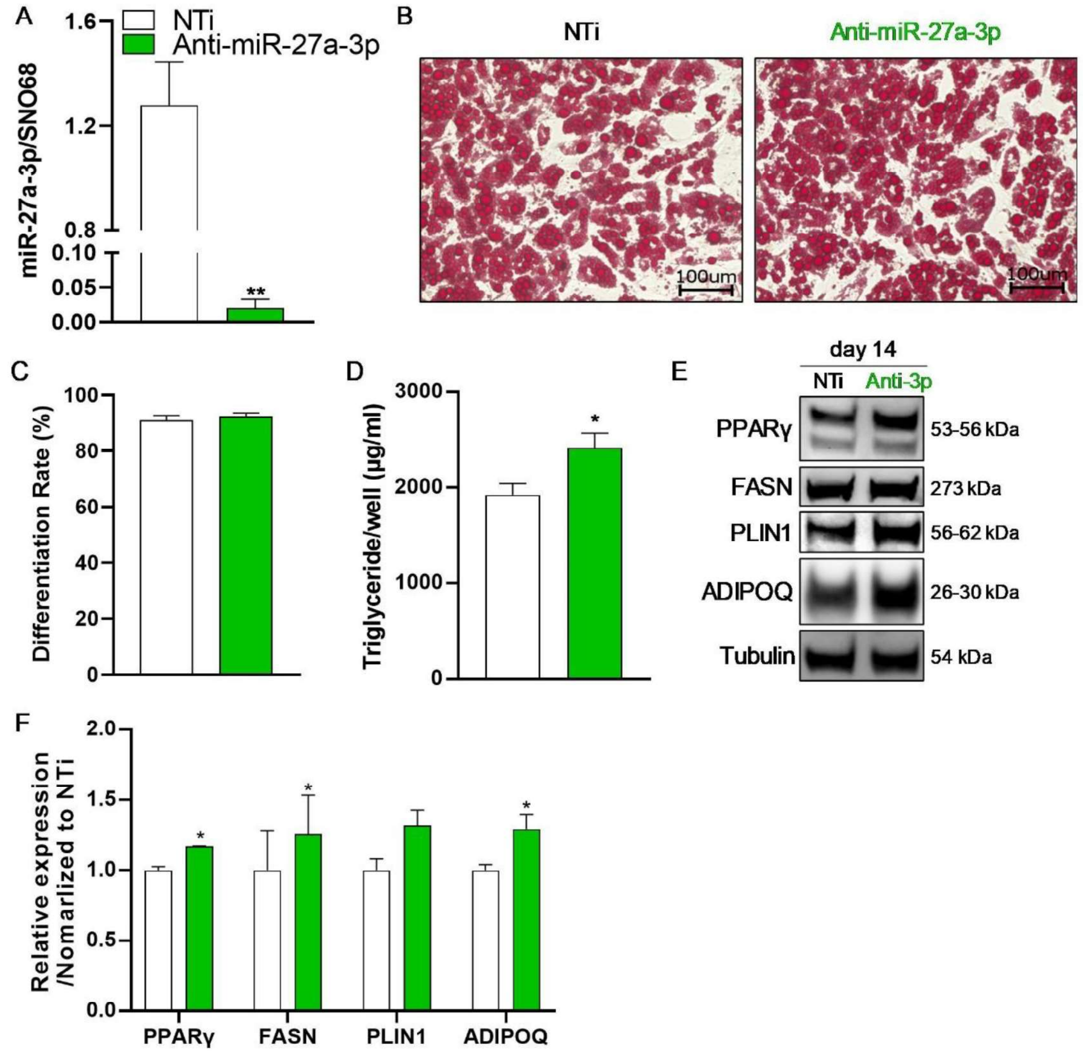

Figure S10. Adipogenic differentiation is enhanced by miR-27a-3p inhibitor in SGBS cells. To assess the effect of miR-27a-3p on SGBS cells, preadipocytes were transfected 48h prior adipogenic induction with miRNA inhibitor or non-target inhibitor (NTi, 50nM). (A) MiRNA expression on day 0 of adipogenic differentiation (48h after transfection quantified by qPCR and related to SNO68). (B) Microphotographs of transfected SGBS cells stained with Oil red O at day 14 of adipogenic differentiation. Lipid droplets are stained in red. (C) Differentiation rate and (D) triglyceride content on day 14 during the differentiation process. (E) One representative Western blot out of three independent experiments after miR-27a-3p inhibition in SGBS cells. (F) Densitometric analysis of three Western blots on day 14 of adipogenesis related to Tubulin. Statistics: results are displayed as mean and SEM of 3 independent experiments. T-test related to NTi; *p<0.05, **p<0.01. SNO68: SNORD68. PPARγ: peroxisome proliferator-activated receptors γ, FASN: fatty acid synthase, PLIN1: perilipin, ADIPOQ: adiponectin. Anti-3p: Anti-miR-27a-3p.

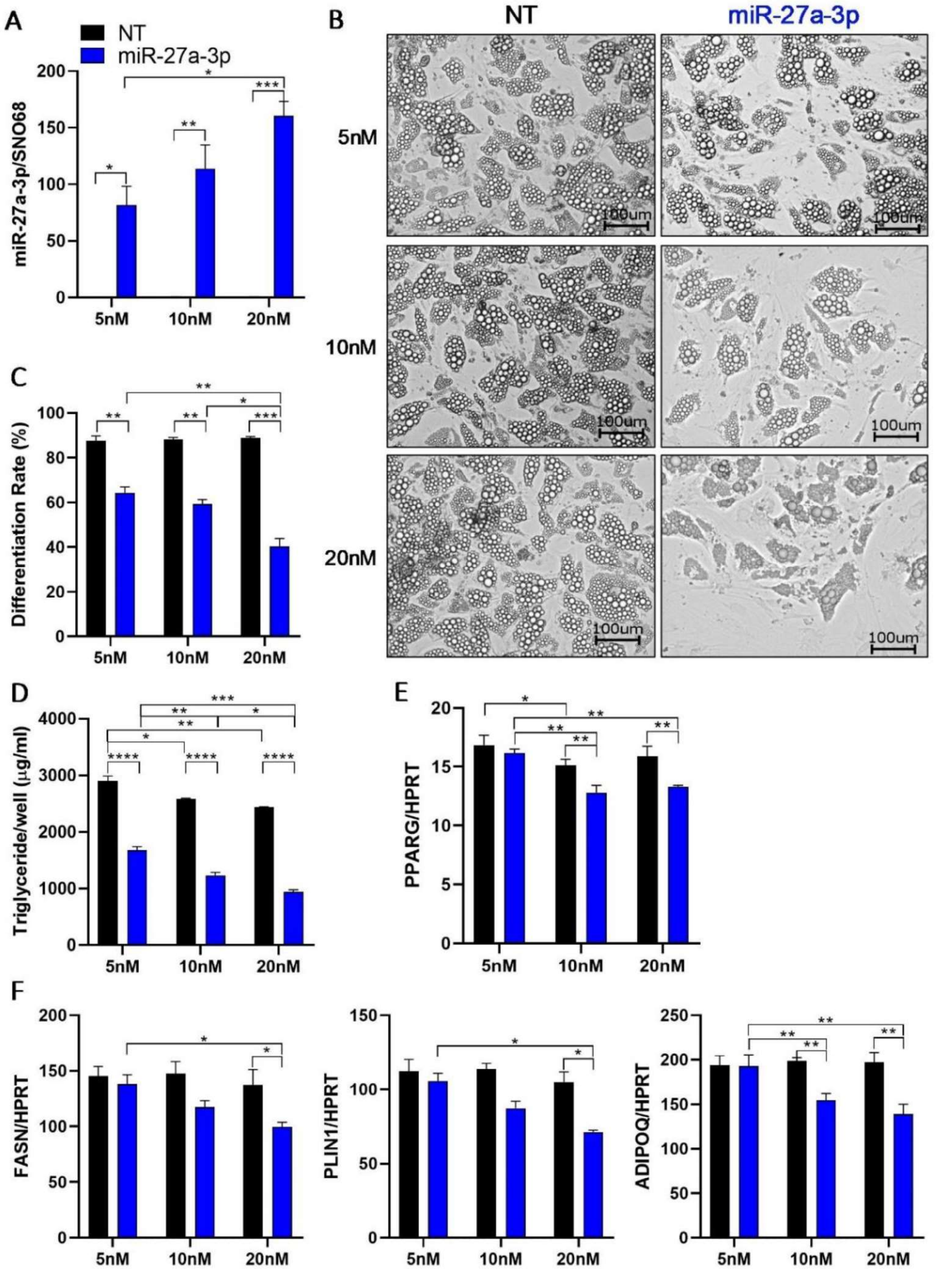

Figure S11: Lower dose of miR-27a-3p can decrease adipogenic differentiation in SGBS cells. To assess the effect of lower concentration of miR-27a-3p on human adipogenesis, SGBS preadipocytes were transfected 48h prior adipogenic induction with miRNA mimics or non-target control (NT) in different concentration (5nM, 10nM, 20nM). **(A)** miRNA expression on day 0 of adipogenic differentiation (48h after transfection quantified by qPCR and related to SNO68). **(B)** Micrographs of transfected SGBS cells on day 14. **(C)** Differentiation rate and **(D)** triglyceride content on day 14. **(E)** mRNA expression of PPARG quantified by qPCR related to HPRT. **(F)** mRNA expression of adipogenic markers Fatty Acid Synthase (FASN), Perilipin (PLIN1) and Adiponectin (ADIPOQ) quantified by qPCR related to HPRT. Statistics: results are displayed as mean and SEM of 3 independent experiments. Two-way ANOVA with Bonferroni correction as marked; *p<0.05, **p<0.01, ***p<0.001; ****p<0.0001. SNO68: SNORD68, HPRT: Hypoxanthine-Guanin-Phosphoribosyltransferase.

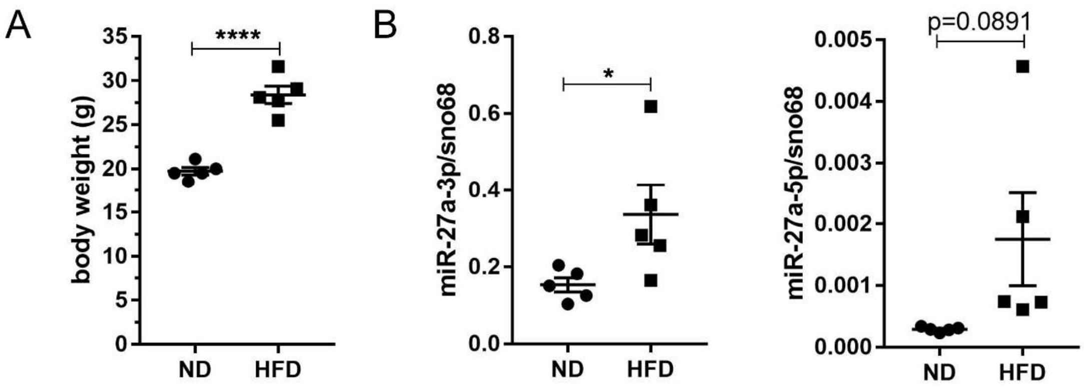

Figure S12: miR-27a-3p and -5p expression is increased in gonadal WAT after 8 weeks of high-fat diet (HFD). Female C57BL/6 J were fed a HFD (D12492n(I) mod. 60 kJ% fat from lard, Ssniff Spezialdiäten, Soest, Germany) or a respective control diet (normal diet, ND, D12450B mod. LS 13 kJ% fat from vegetable oil, Ssniff Spezialdiäten, Soest, Germany). **(A)** Body weight **(B)** miRNA expression quantified by qPCR and related to SNO68. Statistics: results are displayed as mean and SEM of 5 mice per group. Unpaired two-sided t-test ; *p<0.05, ****p<0.0001. SNO68: SNORD68

Table S1. Significant results of the EnrichR analysis for WikiPathway. 724 genes which are predicted as targets of miR-27a-3p by three databases (TargetScan, miRWalk and StarBase) were analyzed using EnrichR [32]. The biological pathway database WikiPathway revealed 60 significantly enriched pathways (adjusted P-value<0.01).

Term	Overlap	Adjusted P-value	Genes
ErbB Signaling Pathway WP673	19/91	1.83×10^{-7}	SHC4, GSK3B, MAP2K4, CAMK2D, PRKCB, GAB1, CBLB, NRG1, FOXO1, NRAS, RPS6KB1, PAK6, ABL2, KRAS, GRB2, SOS1, MAP2K7, PDK1, HBEGF
EGF/EGFR Signaling Pathway WP437	23/162	3.85×10^{-6}	MEF2C, PRKCB, NCOA3, LIMK2, GAB1, CBLB, AP2B1, MAPK14, FOXO1, EPS8, RPS6KA5, CREB1, RPS6KB1, SP1, NEDD4, REPS2, FOSB, SPRY2, GRB2, KRAS, SOS1, MAP3K4, ARF6
Insulin Signaling WP481	22/160	9.30×10^{-6}	MAP2K4, GSK3B, PRKAA2, IRS1, PRKCB, INSR, GAB1, CBLB, SORBS1, FOXO3, MAPK14, FOXO1, RPS6KA5, TBC1D4, RPS6KB1, KIF3A, GRB2, MAP2K7, SOS1, MAP3K4, MAP3K12, ARF6
Sterol Regulatory Element-Binding Proteins (SREBP) signalling WP1982	14/69	1.27×10^{-5}	PRKAA2, HMGCS1, LPL,

			ACLY, CREB1, RNF139, GPAM, SP1, FASN, PPARG, LPIN1, LDLR, KPNB1, PPARGC1B
Signaling Pathways in Glioblastoma WP2261	15/82	1.55×10^{-5}	MAP2K4, IRS1, PRKCB, GAB1, FOXO3, FOXO1, NRAS, CDK6, NF1, SPRY2, MDM4, KRAS, GRB2, MAP2K7, MET
**Adipogenesis WP236**	18/130	5.93×10^{-5}	MEF2C, IRS1, EPAS1, CELF1, LPL, RORA, LIFR, GATA2, FOXO1, KLF7, CREB1, SP1, RARA, ID3, PPARG, IL6ST, LPIN1, LPIN2
Angiopoietin Like Protein 8 Regulatory Pathway WP3915	18/132	5.93×10^{-5}	MAP2K4, GSK3B, THRB, PRKAA2, IRS1, INSR, LPL, CBLB, FOXO3, MAPK14, FOXO1, RPS6KA5, RPS6KB1, FASN, MAP2K7, SOS1, MAP3K4, MAP3K12
TGF-beta Signaling Pathway WP366	18/132	5.93×10^{-5}	SMAD2, MAP2K4, MEF2C, LIMK2, FN1, NUP153, MAPK14, TGFBR1, TGFBR3, SP1, FOSB, GRB2, DCP1A, SOS1, MET, RNF111, ATF3, PDK1
MECP2 and Associated Rett Syndrome WP3584	12/62	7.50×10^{-5}	MEF2C, CREB1, ARHGEF26, SP1,

			HNRNPF, TET3, NF1, TET1, CTCF, NREP, POU3F2, CDON
MAPK Signaling Pathway WP382	25/246	1.26×10^{-4}	CACNA1A, DUSP16, PPP3R1, NRAS, RPS6KA5, MKNK2, MAP2K7, MAP3K4, MAP2K4, MEF2C, GNG12, MAPK14, TGFBR1, CDC25B, CACNB2, MAPKAPK3, TAOK1, RASA2, NF1, RAPGEF2, GRB2, TAB2, KRAS, SOS1, MAP3K12
Nanoparticle-mediated activation of receptor signaling WP2643	8/28	1.50×10^{-4}	NRAS, PXN, ITGA1, FN1, KRAS, GRB2, MAPK14, SOS1
VEGFA-VEGFR2 Signaling Pathway WP3888	24/236	1.53×10^{-4}	MAP2K4, GSK3B, MEF2C, NRP2, EGR3, PRKAA2, PRKCB, PXN, GAB1, PTPRJ, FOXO3, MAPK14, F3, FOXO1, DLL4, RPS6KA5, CREB1, RPS6KB1, GIPC1, PTPN9, GRB2, MAP2K7, HBEGF, ARF6
Energy Metabolism WP1541	10/47	1.53×10^{-4}	GSK3B, PPP3R1, MEF2C, PRKAA2, CREB1, PPARG, FOXO3, MAPK14, FOXO1, PPARGC1B

Wnt Signaling Pathway WP363	10/52	3.69×10^{-4}	GSK3B, CDK6, APC, PRKCB, DVL2, ROR1, PPARG, PIP5K1B, CSNK1G1, LRP6
Oncostatin M Signaling Pathway WP2374	11/65	4.34×10^{-4}	CREB1, IRS1, PRKCB, PXN, LIFR, KRAS, GRB2, IL6ST, SOS1, MAPK14, LDLR
Pathways Affected in Adenoid Cystic Carcinoma WP3651	11/65	4.34×10^{-4}	FBXW7, NFIB, KMT2C, NSD1, ATRX, JMJD1C, ARID5B, BCOR, BCORL1, FOXO3, FOXP2
Integrated Breast Cancer Pathway WP1984	17/151	7.21×10^{-4}	SMAD2, IRS1, NCOA3, MYCBP2, PHB, TFPI, FOXO1, TGFBR1, CDC25B, HIPK2, EDAR, ITPKC, CASP8, CREB1, SP1, NF1, KRAS
Ras Signaling WP4223	19/184	8.11×10^{-4}	SHC4, KSR1, PRKCB, INSR, GAB1, RASAL2, GNG12, NRAS, RASSF5, RASA2, NF1, ABL2, PAK6, GRB2, KRAS, SOS1, RGL2, MET, ARF6
Focal Adhesion-PI3K-Akt-mTOR-signaling pathway WP3932	26/303	8.54×10^{-4}	PHLPP2, GSK3B, PRKAA2, CAB39, CSF1, EPAS1, IRS1, FOXO3, FOXO1, NRAS, RELN, ITGB8, INSR, FN1, GNG12, KITLG, CREB1, COL5A1,

			RPS6KB1, RAB14, LPAR6, GRB2, KRAS, ITGA5, SOS1, MET
Epithelial to mesenchymal transition in colorectal cancer WP4239	17/159	0.0012	SMAD2, MAP2K4, GSK3B, FZD3, NRP2, FZD4, EIF5A2, FN1, NR2C2, MAPK14, TGFBR1, LRP6, DLL4, GRB2, KRAS, ITGA5, SOS1
Brain-Derived Neurotrophic Factor (BDNF) signaling pathway WP2380	16/144	0.0012	SHC4, GSK3B, MEF2C, PRKAA2, KSR1, CRTC1, IRS1, FOXO3, MAPK14, MARCKS, RPS6KA5, CREB1, APC, RPS6KB1, GRB2, CDK5R1
Extracellular vesicle-mediated signaling in recipient cells WP2870	7/30	0.0013	SMAD2, TGFBR3, NRAS, APC, KRAS, MET, TGFBR1
Genotoxicity pathway WP4286	10/63	0.0013	ACTA2, ITPKC, BTG2, DAAM1, RBM12B, GXYLT1, RAPGEF2, CBLB, SMAD5, E2F7
Leptin signaling pathway WP2034	11/76	0.0013	GSK3B, CREB1, PRKAA2, RPS6KB1, IRS1, SP1, PDE3B, GRB2, SOS1, MAPK14, FOXO1
Factors and pathways affecting insulin-like growth factor (IGF1)-Akt signaling WP3850	7/31	0.0013	SMAD2, GSK3B, MSTN, RPS6KB1, IRS1, ACVR2B, PDK1
Transcription factor regulation in	6/22	0.0013	CREB1, IRS1,

adipogenesis WP3599			INSR, PPARG, LPIN1, FOXO1
Insulin signalling in human adipocytes (diabetic condition) WP3635	4/8	0.0013	TBC1D4, RPS6KB1, IRS1, INSR
Insulin signalling in human adipocytes (normal condition) WP3634	4/8	0.0013	TBC1D4, RPS6KB1, IRS1, INSR
White fat cell differentiation WP4149	7/32	0.0015	CREB1, IRF4, RARA, RORA, PPARG, GATA2, FOXO1
Thermogenesis WP4321	13/108	0.0017	KDM3A, PRKAA2, ADCY3, MAPK14, ARID1B, ADCY6, NRAS, CREB1, RPS6KB1, GRB2, PPARG, KRAS, SOS1
DNA Damage Response (only ATM dependent) WP710	13/110	0.0020	GSK3B, IRS1, INSR, FOXO3, NRAS, APC, DVL2, GRB2, KRAS, SOS1, LDLR, MAP3K4, PDK1
BDNF-TrkB Signaling WP3676	7/34	0.0020	NRAS, CREB1, RPS6KB1, GAB1, KRAS, GRB2, SOS1
B Cell Receptor Signaling Pathway WP23	12/97	0.0021	GSK3B, MEF2C, CREB1, PRKCB, IRF4, GAB1, PIP5K1B, GRB2, SOS1, MAPK14, FOXO1, GTF2I
Aryl Hydrocarbon Receptor WP2586	8/46	0.0023	RET, NRAS, NF1, LPL, CYP1B1, NCOA7, KRAS, NFE2L2
Kit receptor signaling pathway WP304	9/59	0.0026	KITLG, RPS6KB1, PRKCB, MITF, GRB2, SOS1,

			FOXO3, SOCS6, MAPK14
Canonical and Non-Canonical TGF-B signaling WP3874	5/17	0.0026	SMAD2, GREM1, LOX, MAPK14, TGFBR1
ESC Pluripotency Pathways WP3931	13/116	0.0028	ACVR1, GSK3B, FZD3, FZD4, GAB1, LIFR, SMAD5, LRP6, APC, DVL2, GRB2, IL6ST, SOS1
Regulation of Actin Cytoskeleton WP51	15/150	0.0034	PPP1R12A, PXN, ITGA1, FN1, MSN, GNG12, ENAH, GNA13, NRAS, APC, PAK6, PIP5K1B, KRAS, SOS1, MYH10
Nuclear Receptors WP170	7/38	0.0034	THRB, RARA, RORA, NR1D2, PPARG, ROR1, NR2C2
Hippo-Merlin Signaling Dysregulation WP4541	13/120	0.0036	PPP1R12A, INSR, ITGA1, LIN28B, NRAS, CDH11, CDH24, ITGB8, PAK6, KRAS, ITGA5, TEAD1, MET
Breast cancer pathway WP4262	15/154	0.0041	SHC4, GSK3B, FZD3, NCOA3, LRP6, DLL4, NRAS, CDK6, APC, RPS6KB1, SP1, DVL2, GRB2, KRAS, SOS1
TNF alpha Signaling Pathway WP231	11/92	0.0041	MAP2K4, NRAS, CASP8, APAF1, KSR1, TNFAIP3, KRAS, TAB2, GRB2, SOS1, MAP2K7

MAPK Cascade WP422	6/29	0.0042	MAP2K4, NRAS, KRAS, MAP2K7, MAPK14, MAP3K12
AGE/RAGE pathway WP2324	9/66	0.0046	SMAD2, CASP8, IRS1, PRKCB, SP1, INSR, MSN, MAPK14, FOXO1
Association Between Physico-Chemical Features and Toxicity Associated Pathways WP3680	9/66	0.0046	GSK3B, MAP2K4, FZD3, DAAM1, APC, FZD4, FN1, GRB2, SOS1
Non-small cell lung cancer WP4255	9/66	0.0046	NRAS, CDK6, PRKCB, RASSF5, KRAS, GRB2, SOS1, FOXO3, PDK1
PI3K-AKT-mTOR signaling pathway and therapeutic opportunities WP3844	6/30	0.0047	GSK3B, NRAS, KRAS, FOXO3, FOXO1, PDK1
Hematopoietic Stem Cell Differentiation WP2849	8/55	0.0056	KITLG, THRB, CSF1, FOSB, IKZF1, GATA2, CBFA2T3, RUNX1
Neovascularisation processes WP4331	5/21	0.0056	SMAD2, DLL4, EPHB2, SMAD5, TGFBR1
IL-6 signaling pathway WP364	7/43	0.0058	GSK3B, MAP2K4, RPS6KB1, GAB1, GRB2, IL6ST, SOS1
Mesodermal Commitment Pathway WP2857	14/147	0.0063	ACVR1, SMAD2, CRTC1, FZD4, ARID5B, TET1, HTT, ACVR2B, ACVR2A, ADAM19, CSRP2, BCORL1, TEAD1, NFE2L2
Estrogen Receptor Pathway WP2881	4/13	0.0063	GPAM, SP1, PDK4, CYP1B1
Wnt Signaling WP428	12/115	0.0063	GSK3B, SFRP1,

			PPP3R1, FZD3, CAMK2D, DAAM1, APC, PRKCB, DVL2, PRICKLE2, ROR1, LRP6
Type II diabetes mellitus WP1584	5/22	0.0064	GK, IRS1, INSR, CACNA1A, SOCS4
miRNA regulation of prostate cancer signaling pathways WP3981	6/33	0.0068	GSK3B, PLCL2, KRAS, GRB2, SOS1, FOXO1
Prostaglandin Synthesis and Regulation WP98	7/45	0.0069	PTGFR, EDNRA, PTGER3, MITF, PPARG, PTGFRN, PPARGC1B
MET in type 1 papillary renal cell carcinoma WP4205	8/59	0.0075	NRAS, GAB1, PAK6, KRAS, GRB2, STRN, SOS1, MET
Signaling of Hepatocyte Growth Factor Receptor WP313	6/34	0.0075	PXN, ITGA1, GAB1, GRB2, SOS1, MET
p38 MAPK Signaling Pathway WP400	6/34	0.0075	MAP2K4, RPS6KA5, CREB1, GRB2, MAPK14, TGFBR1
PI3K-Akt Signaling Pathway WP4172	24/340	0.0089	PHLPP2, GSK3B, PRKAA2, CSF1, IRS1, INSR, ITGA1, FN1, GNG12, FOXO3, NRAS, KITLG, RELN, G6PC3, CDK6, CREB1, RPS6KB1, LPAR6, ITGB8, GRB2, KRAS, ITGA5, SOS1, MET
